# Activation of the yeast Retrograde Response pathway by adaptive laboratory evolution with S-(2-aminoethyl)-L-cysteine reduces ethanol and increases glycerol during winemaking

**DOI:** 10.1186/s12934-024-02504-z

**Published:** 2024-08-20

**Authors:** Víctor Garrigós, Cecilia Picazo, Emilia Matallana, Agustín Aranda

**Affiliations:** Institute for Integrative Systems Biology (I2SysBio), Universitat de València-CSIC, C/ Catedrático Agustín Escardino 9, 46980 Paterna, Valencia Spain

**Keywords:** Adaptive laboratory evolution, *Saccharomyces cerevisiae*, Retrograde Response pathway, Ethanol, Glycerol, Acetic acid

## Abstract

**Background:**

Global warming causes an increase in the levels of sugars in grapes and hence in ethanol after wine fermentation. Therefore, alcohol reduction is a major target in modern oenology. Deletion of the *MKS1* gene, a negative regulator of the Retrograde Response pathway, in *Saccharomyces cerevisiae* was reported to increase glycerol and reduce ethanol and acetic acid in wine. This study aimed to obtain mutants with a phenotype similar to that of the *MKS1* deletion strain by subjecting commercial *S. cerevisiae* wine strains to an adaptive laboratory evolution (ALE) experiment with the lysine toxic analogue S-(2-aminoethyl)-L-cysteine (AEC).

**Results:**

In laboratory-scale wine fermentation, isolated AEC-resistant mutants overproduced glycerol and reduced acetic acid. In some cases, ethanol was also reduced. Whole-genome sequencing revealed point mutations in the Retrograde Response activator Rtg2 and in the homocitrate synthases Lys20 and Lys21. However, only mutations in Rtg2 were responsible for the overactivation of the Retrograde Response pathway and ethanol reduction during vinification. Finally, wine fermentation was scaled up in an experimental cellar for one evolved mutant to confirm laboratory-scale results, and any potential negative sensory impact was ruled out.

**Conclusions:**

Overall, we have shown that hyperactivation of the Retrograde Response pathway by ALE with AEC is a valid approach for generating ready-to-use mutants with a desirable phenotype in winemaking.

**Supplementary Information:**

The online version contains supplementary material available at 10.1186/s12934-024-02504-z.

## Background

The wine industry must be constantly evolving to keep up with changing trends. Nowadays, wine consumers are demanding new sensory profiles, and markets are driven by higher aromatic intensity, freshness, full-bodied and ripe fruit flavour profiles and lower alcohol content [42, 56]. However, current climate change leads to the overripening of grapes at harvest, increasing the sugar content and lowering the acidity, hence producing high-alcohol wines that lack freshness [29, 39]. Fermentation of grape juice by yeasts can be targeted to address all these issues. The most frequently used species in the industry is *Saccharomyces cerevisiae* due to its fermentative power and adaptation to the winemaking conditions, resulting in rapid and predictable fermentation. The success of *S. cerevisiae* lies in its ability to adapt metabolically to the changing environment of the industrial processes to which it is subjected [37]. Nutrient signalling pathways are the main molecular systems responsible for controlling growth and stress response by sensing the presence or absence of nutrients outside and inside the cell [8]. The main pathways in *S. cerevisiae*, common to all eukaryotes, are the TOR pathway (which senses mainly nitrogen) and Protein Kinase A (that responds to the presence of glucose).

A phenomic analysis of mutations in nutrient signalling pathways in a haploid wine yeast strain revealed the key relevance of PKA and TORC1 during winemaking [52]. Under the same conditions, deletion of *MKS1* led to an increase in glycerol and a decrease in ethanol and acetic acid [19]. This phenotype was consistent in several commercial wine strains, as well as in brewer's and baker's yeasts [19]. *MKS1* encodes a repressor of the Retrograde Response (RR), a signalling pathway that communicates mitochondrial dysfunction to the nucleus (reviewed in Jazwinski [26]). When mitochondrial function is impaired, the complex formed by the transcription factors Rtg1 and Rtg3 translocates to the nucleus and triggers the induction of a broad array of target genes [48]. The subcellular location of the Rtg1/3 complex is controlled by the repressor Mks1 and the activator Rtg2 [12, 34]. RR-targets include genes encoding mitochondrial and peroxisome enzymes that divert cytosolic pyruvate and acetyl-CoA to citrate and then to α-ketoglutarate, a precursor for glutamate/glutamine and lysine biosynthesis (reviewed in Jazwinski [26]). Due to its role in nitrogen metabolism, RR is repressed by the TOR complex in conditions of abundance through its negative regulator Mks1 [9]. Thus, the *MKS1* deletion mutant exhibits hyperactivated Retrograde signalling and increased expression of RR targets and most of the *LYS* genes involved in lysine biosynthesis [12], making it more tolerant to a toxic analogue of lysine called S-aminoethyl-L-cysteine (AEC,also called thialysine [16].

**Fig. 1:**
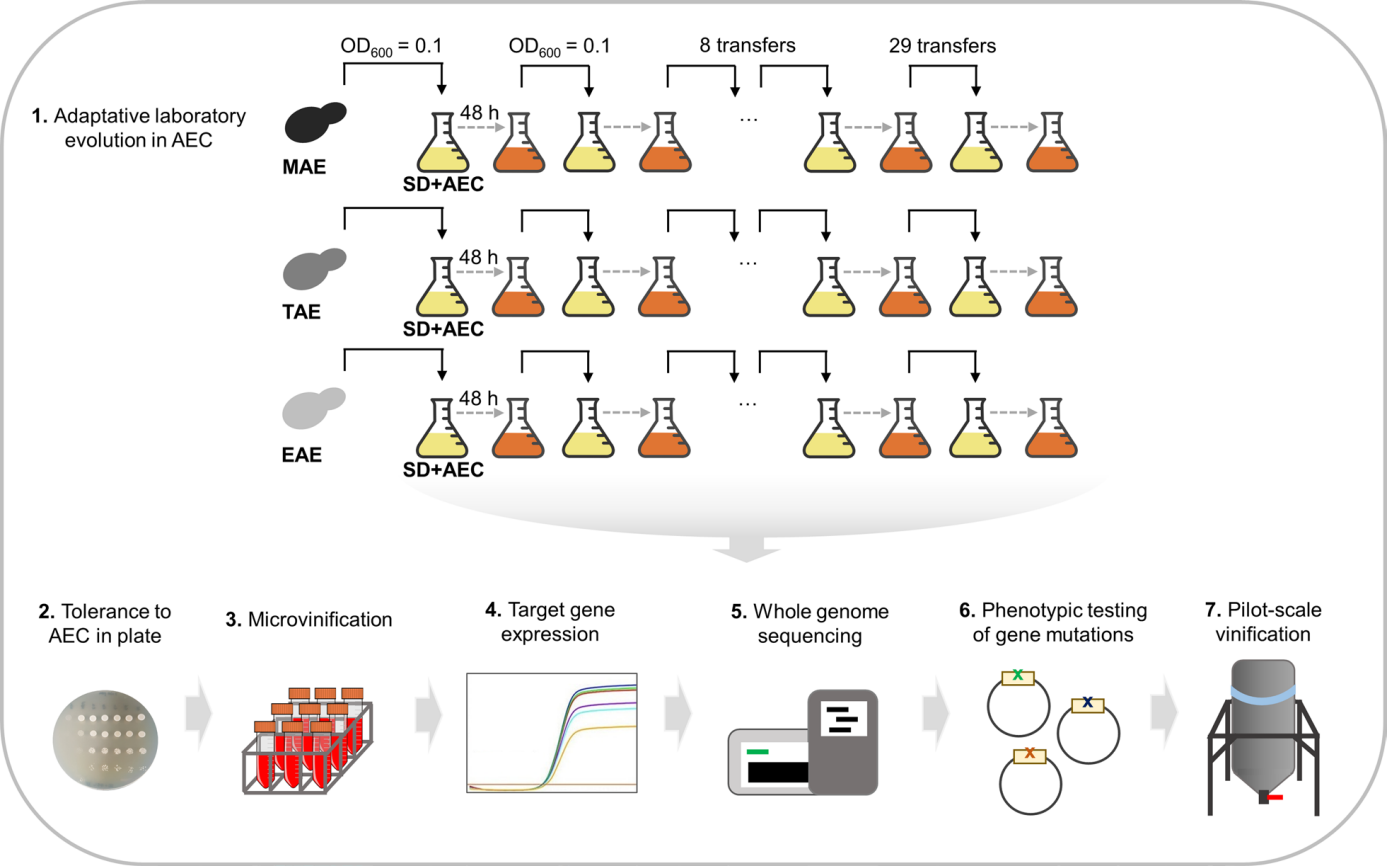
Overview of the experimental design described in this study. Steps and experimental procedures for generating new AEC-resistant mutants with improved winemaking phenotypes from *S. cerevisiae* wine strains. Adaptive laboratory evolution – Commercial *S. cerevisiae* wine strains MAE, TAE and EAE were serially transferred (up to 29 transfers) into fresh SD media supplemented with 35 mg/L of AEC. Tolerance to AEC in plate – Individual clones isolated from evolved populations were tested for resistance to AEC by spot-analysis to confirm their suitability for further steps. Microvinification – The winemaking phenotype of clones isolated from the evolved populations was analysed by laboratory-scale fermentations in natural grape must. Target gene expression—The expression of Retrograde Response target genes was analysed in those isolated clones that showed a better phenotype in winemaking. Whole-genome sequencing – The whole genomes of selected clones were sequenced to identify possible mutations causing the phenotype of interest. Phenotypic testing of gene mutations—The identified mutations were cloned into an expression vector in yeast to confirm causality. Pilot-scale vinification—Wine fermentations were scaled up to verify the suitability of an isolated mutant in a relevant environment

Nutrient signalling pathways are good targets for improving the production of metabolites of interest. For example, genetic manipulation of TOR components in wine yeasts or chemical inhibition by the herbicide glufosinate have been used to increase glycerol production during winemaking [54, 55]. In *S. cerevisiae*, glycerol plays a major role in redox homeostasis and osmotic stress resistance [2] and contributes positively to the quality of wine [63]. Much work has been done on genetic manipulation to redirect metabolism towards increased glycerol production and thus away from ethanol accumulation [10, 38, 40, 45, 57] reviewed in [22]. Due to redox unbalances, this type of manipulation leads to an undesired increase in acetic acid, and additional genetic manipulations are necessary to reduce it [4, 14]. However, the use of Genetically Modified Organisms (GMOs) for the food industry still has the main disadvantage of consumer rejection, in addition to strict production and labelling regulations. Consequently, there is great interest in using alternative approaches to improve the properties of wine yeast strains without genetic modification. Adaptive Laboratory Evolution (ALE), which is based on long-term adaptation under environmental or metabolic constraints, is a non-GMO alternative and has been described as a powerful tool in modern industrial biotechnology [47]. ALE has been successfully used in wine yeast to reduce ethanol production and increase glycerol levels by applying osmotic pressure [50], but also to achieve higher fermentation rates and enhanced production of aroma compounds [3]. Recently, it has been reported as a promising strategy to obtain strains that do not increase volatile acidity during winemaking under aerobic conditions [23]. In this work, we described a workflow (Fig. 1) to generate AEC-resistant *S. cerevisiae* wine strains by ALE and to select those that resemble the previously characterized *mks1*∆ phenotype in winemaking. Several evolved strains achieved reduced acetic acid production, increased glycerol levels and reduced ethanol in laboratory-scale vinifications, and mutations in *RTG2* were identified as responsible. One of them was further investigated for reproducing this relevant industrial phenotype at the pilot scale.

## Materials and methods

### Strains and culture conditions

The *Saccharomyces cerevisiae* commercial wine strains MAE, TAE and EAE (Lallemand, Co., Canada) were used for adaptive laboratory evolution (ALE) experiments, and the haploid wine strain C9 [59] was used for genetic manipulation. The complete list of strains used in this work is available in Additional file 1.

For standard propagation and genetic manipulation, yeasts were cultured in YPD media (10 g/L yeast extract, 20 g/L peptone and 20 g/L glucose) at 30 °C and 180 rpm. For the selection of yeast strains with the *kan*MX dominant marker, YPD medium supplemented with 200 mg/L geneticin was used. Minimal medium SD (1.7 g/L yeast nitrogen base without amino acids, 5 g/L ammonium sulphate, 20 g/L glucose) or SC (the same as SD but supplemented with the indicated amount of drop-out powder (Formedium)) supplemented with 2 g/L cycloheximide was used to select the transformants with cycloheximide resistance. The solid media were prepared by the addition of 20 g/L agar before sterilization.

ALE experiments and growth tests were performed in SD media supplemented with 35 mg/L of 2-aminoethyl-L-cysteine (AEC), denoted as SD + AEC. Microfermentation experiments were conducted in red grape juice (Bobal variety) sterilized overnight with 500 mg/L dimethyl dicarbonate in cold. For pilot-scale fermentations, 50 kg of tempranillo grapes were used at Vitec, Wine Technology Centre (Falset, Spain).

The *Escherichia coli* NZYα strain (NzyTech) was used to maintain and amplify the cloned plasmids. *E. coli* cells were propagated in LB media (10 g/L of tryptone, 5 g/L of yeast extract and 5 g/L of NaCl) supplemented with 100 mg/L of ampicillin to maintain plasmids at 37 °C and 220 rpm.

### Adaptive laboratory evolution

The *S. cerevisiae* industrial wine strains MAE, TAE and EAE were used as the original parental strains for the ALE experiments. For ALE, a single colony of the parental strain was inoculated into 5 mL of SD media and grown overnight. This culture was used to inoculate 125 mL flasks containing 25 mL of SD + AEC (35 mg/L) at an initial OD_600_ of 0.1. The cultures were grown at 30 °C and 180 rpm until they reached the stationary phase before being transferred into fresh medium every 2–3 days at an initial OD_600_ value of 0.1. The number of generations through evolution was calculated by the following equation: n = log_2_ (N_0_ / N_t_), where n is the number of generations, N_0_ is the initial OD_600_ and N_t_ is the OD_600_ at time t [62]. After 8 and 29 transfers, cryostocks of the cultures were prepared, and single clones were isolated by plating 1 µL of the culture on SD + AEC agar. Interdelta analysis of the randomly selected colonies was conducted according to Legras and Karst [30] to determine whether there was any contamination.

### Microfermentation experiments

For the microvinification experiments, cells from 2-day cultures in YPD medium were inoculated at an OD_600_ value of 0.1 into conical centrifuge tubes with 30 mL of red grape juice (Bobal variety), a gift from Bodegas Murviedro (Requena, Spain). Fermentations were performed at 24 °C with low shaking (50 rpm). The vinification process was followed by taking aliquots of the supernatant every 2–3 days and measuring the consumption of reducing sugars with DNS (dinitro-3,5-salicylic acid) according to Miller’s method [46]. The supernatant was used for metabolite measurement at the end of fermentation. Glycerol and acetic acid were measured with commercial kits (Megazyme Ltd., Bray, Ireland). The enzymatic quantification of ethanol was performed by spectrophotometric detection at 340 nm of NADH formed during the oxidation of ethanol to acetaldehyde by the enzyme alcohol dehydrogenase. The assay was performed in 0.2 M glycine—0.3 M Tris buffer (pH 9.7) supplemented with 2 mM NAD^+^ and 20 U/mL yeast alcohol dehydrogenase in a final volume of 1 mL, and 200 μL of sample was added (appropriate dilution).

### Pilot-scale fermentations

Pilot-scale fermentations were performed at Vitec’s experimental cellar (Falset, Spain). The selected yeast strains were grown in liquid YPD for 48 h at 28 °C and then transferred to Pyrex bottles for growth in 1 L of YPD media. From this volume, total yeast was counted by optic microscopy to inoculate at a concentration of 2 × 10^6^ cells/mL the non-sterile must from 50 kg of Tempranillo grapes. Vinifications were carried out in triplicate and independent vats. Alcoholic fermentation was monitored by daily control of density and temperature and by studying the total and viable yeast population. At the end of fermentation, aliquots of the different fermentations were transferred to YPD plates to control the implantation of the inoculated strain, and the following parameters were analysed: density, sugars (D-glucose/D-fructose), alcoholic strength, total tartaric acidity, volatile acidity, pH, L-malic acid, and glycerol. The analyses were performed according to the protocols established by the Compendium of International Methods of Wine and Must Analysis OIV (2011).

Aroma and taste analysis were carried out by a panel of 10 wine experts who are part of Vitec's accredited tasting panel. The samples were presented simultaneously in a different order for each expert based on a Latin square experimental design. All the samples were served at room temperature and the panellists were not informed about the nature of the samples to be evaluated.

### Plasmid construction

The plasmids and primers used herein are listed in Additional files 2 and 3, respectively. For cloning the different alleles of the *RTG2*, *LYS20* and *LYS21* genes, each gene containing its promoter and terminator was PCR-amplified from the genomic DNA of the parental and selected evolved strains using Phusion DNA polymerase (Thermo Scientific). The primers RTG2-X/RTG2-B, LYS20-X/LYS20-B, and LYS21-X/LYS21-B were used for *RTG2*, *LYS20* and *LYS21* amplification, respectively. PCR products and the pCUP1pNuiHA kanMX CEN plasmid were digested with the restriction enzymes XhoI and BamHI, gel-checked and purified using mi-PCR Purification Kit (Metabion). pCUP1pNuiHA kanMX CEN was a gift from Nils Johnsson (Addgene plasmid #131168; http://n2t.net/addgene:131168; RRID:Addgene_131168) [13]. Then, the ligation reactions were carried out using T4 DNA Ligase Kit (NzyTech). The *E. coli*-positive transformants were selected and the plasmids were PCR-checked and sequenced.

### Yeast genetic manipulation

The recyclable *kan*MX selection marker from plasmid pUG6 [24] was used to perform gene disruptions. This marker contains flanking loxP sites to excise it by employing the Cre recombinase from plasmid YEp-cre-cyh [11]. Yeast cells were transformed by the PEG/LiAc method according to [21]. Routinely, 200–500 ng of plasmid DNA and 0.5–1 µg of linear DNA were used per transformation. All transformants were selected on the corresponding selective agar media.

### Relative gene expression level quantification by real-time PCR

To quantify the relative expression levels of the indicated genes, yeast cells were first cultivated in 5 mL of YPD media overnight and transferred to 50 mL of fresh YPD media. Then, they were cultured to an OD_600_ of 0.6–0.8. The cells were harvested, and total RNA was extracted [6]. The obtained RNA was reverse transcribed using NZY First-Strand cDNA Synthesis Kit (Nzytech). Described pair of primers CIT2f/CIT2r, DLD3f/DLD3r and ACT1f/ACT1r [49] were used to amplify *CIT2* and *DLD3*, and the housekeeping gene (*ACT1*) was used as an internal control. Quantitative PCR using NZYSpeedy qPCR Green Master Mix (Nzytech) was performed with a QuantStudio 3 instrument following the manufacturer’s instructions. Each reaction was carried out in triplicate, and the reported Ct value was reported as the average of triplicate samples. Transcript levels were calculated using the 2^−ΔΔCt^ method [35].

### DNA extraction and sequencing

YeaStar Genomic DNA Kit (Zymo Research) was used to extract genomic DNA from 5 mL of overnight yeast YPD culture using the protocol recommended by the manufacturer. The quality and concentration of the extracted DNA were assessed with a NanoDrop One spectrophotometer (Thermo Scientific). Sequencing, library preparation and subsequent bioinformatics analysis were carried out by the company Novogene. The genomic DNA was randomly sheared into short fragments. The obtained fragments were end repaired, A-tailed and further ligated with Illumina adapter. The fragments with adapters were PCR amplified, size selected, and purified. The library was checked with Qubit and real-time PCR for quantification and bioanalyzer for size distribution detection. The quantified libraries were pooled and sequenced on the Illumina HiSeq^™^ platform. The data recorded in the image files were first transformed to sequence reads by base calling with CASAVA software. The effective sequencing data were aligned with the reference sequences through BWA software (parameters: mem -t 4 -k 32 -M), and the mapping rate and coverage were determined according to the alignment results. Data mapping was performed against the S288c genome (GCF_000146045.2). The mapping rate, depth and coverage statistics are described in Additional file 4, together with the accession numbers for the genomes of the evolved strains deposited in NCBI. This table contains the unique SNPs and InDels of the evolved strains, with the mutations of interest highlighted. Non-synonymous polymorphisms were identified in the CDS regions of selected genes by visual comparison to the parental strains. Mutations in both the parental and evolved strains were excluded from further analysis.

## Results

### Adaptive laboratory evolution of *S. cerevisiae* wine strains and mutant screening

We have previously demonstrated that the deletion of the Retrograde Response repressor *MKS1* increases glycerol and reduces acetic acid production in *S. cerevisiae* wine strains under winemaking conditions [19]. After these results, our interest focused on obtaining ready-to-use strains with the same phenotype but without genetic manipulation. Deletion of *MKS1* has been reported to promote lysine biosynthesis, resulting in increased tolerance to its toxic analogue S-(2-aminoethyl)-L-cysteine (AEC), also called thialysine [16]. This phenotype was also found to be true in wine strains of *S. cerevisiae* [19]. Taking advantage of this phenotype, an adaptive laboratory evolution experiment was designed to generate evolved populations of three commercial *S. cerevisiae* wine strains with increased tolerance to thialysine. The ALE experiment was planned for three different strains to prove that is a reliable and reproducible method that could be applied to any yeast of interest. First, the native tolerance of these strains to AEC was determined by measuring growth after 48 h in SD media supplemented with different concentrations of this compound. A concentration of 35 mg/L AEC was selected for ALE as it reduced yeast growth by ~ 50% (data not shown), thus leaving room for improvement without completely inhibiting yeast growth.

**Fig. 2:**
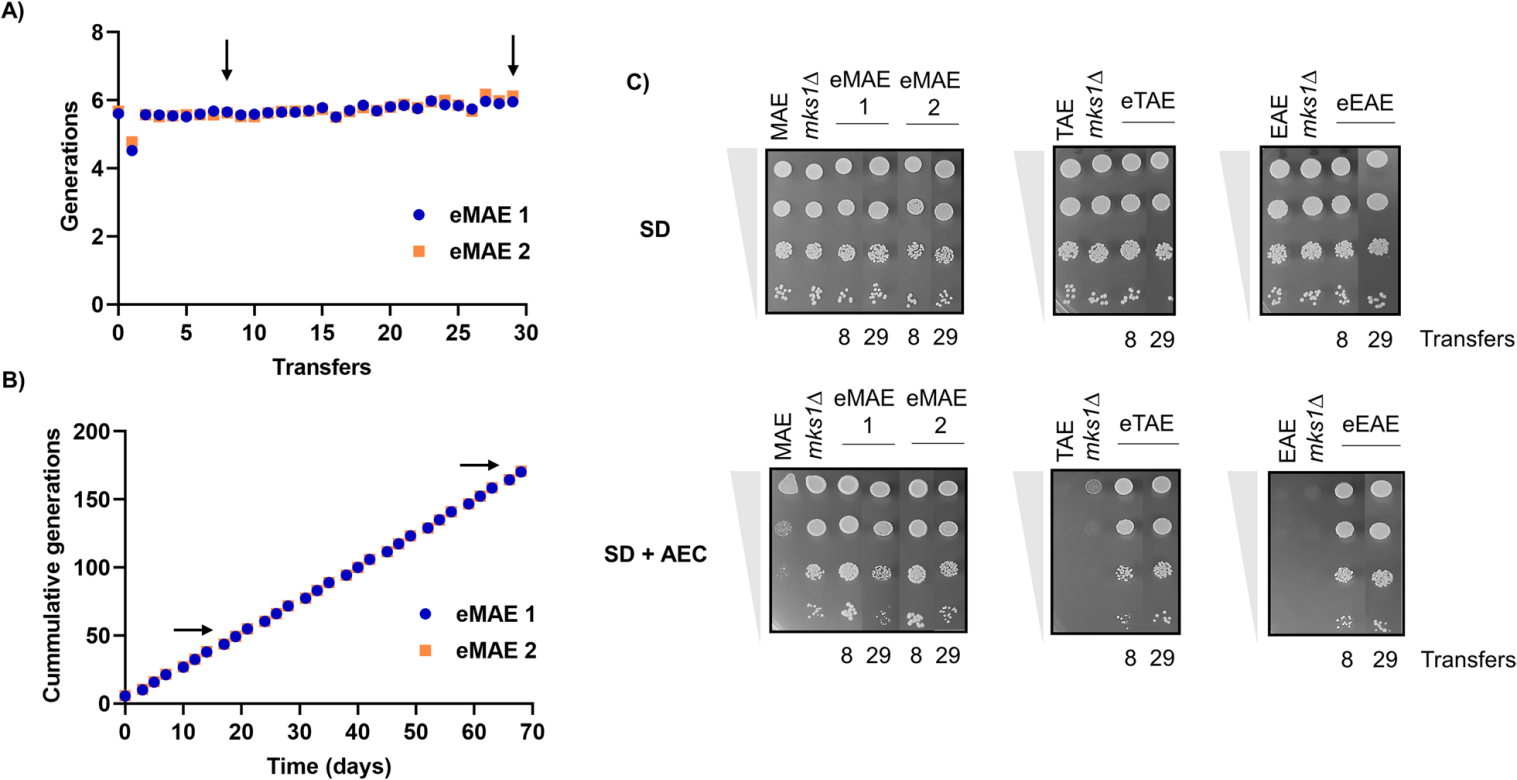
Adaptive laboratory evolution experiments and AEC resistance of individual clones isolated from each evolved population were monitored. **A** Generations obtained in each of the transfers performed during the directed evolution of the two replicates (eMAE 1, eMAE 2) of the MAE strain and **B** accumulated generations over the course of the experiment. The black arrows indicate 8 and 29 transfers, the time points at which individual clones were isolated from the evolved population. **C** Spot growth analysis to test AEC tolerance of several clones isolated from MAE, TAE and EAE evolution after 8 and 29 transfers. A 5 μl volume of each serially diluted culture (from 10^–1^ to 10^–4^) was spotted on SD plates supplemented or not with 35 mg/L of AEC. All plates were incubated at 30 °C for 48 h. Information regarding the progress of the adaptive evolution experiment of strains TAE and EAE, as well as the thialysine resistance of each isolated clone, can be found in Additional file 1

Initially, the ALE experiment in SD + AEC was conducted for the MAE strain. Two single colonies were used as seeds for two independent replicates of the experiment (eMAE 1, eMAE 2). Due to the rapid adaptation of the strain under the studied conditions, single colonies of each replicate were isolated on SD + AEC (35 mg/L) plates after 8 transfers. The ALE experiments were stopped after 29 transfers due it was found that there was no change in maximum cell density (Fig. 2A). In both replicates, a similar number of cumulative generations was achieved over time (Fig. 2B). The same strategy was followed for the TAE and EAE strains, and the experiment was performed in parallel with a single replicate of each strain. Adaptation of EAE was faster and reached a higher number of cumulative generations than TAE (Additional file 5A and 5B).

Within each ALE experiment, five random individual clones were picked from culture plates and increased tolerance to AEC was confirmed. Figure 2C shows a spot analysis of one clone from each directed evolution experiment, after 8 and 29 transfers, compared to its parental strain and its respective *MKS1* null mutant. After 8 transfers, it was already observed that these clones had greater tolerance to AEC than their parental strains. This phenotype was compatible with hyperactivation of the Retrograde Response pathway, as these strains were even more tolerant than the *MKS1* mutants. This phenotype was also observed for the five tested clones from each ALE experiment (Additional file 5**C**).

### Phenotypic characterization of the evolved mutants during wine fermentation

In order to study whether the evolved clones show a phenotype compatible with the *MKS1* null mutant in wine fermentation [19], microvinification experiments were conducted on red grape must with the most promising clones.

**Fig. 3:**
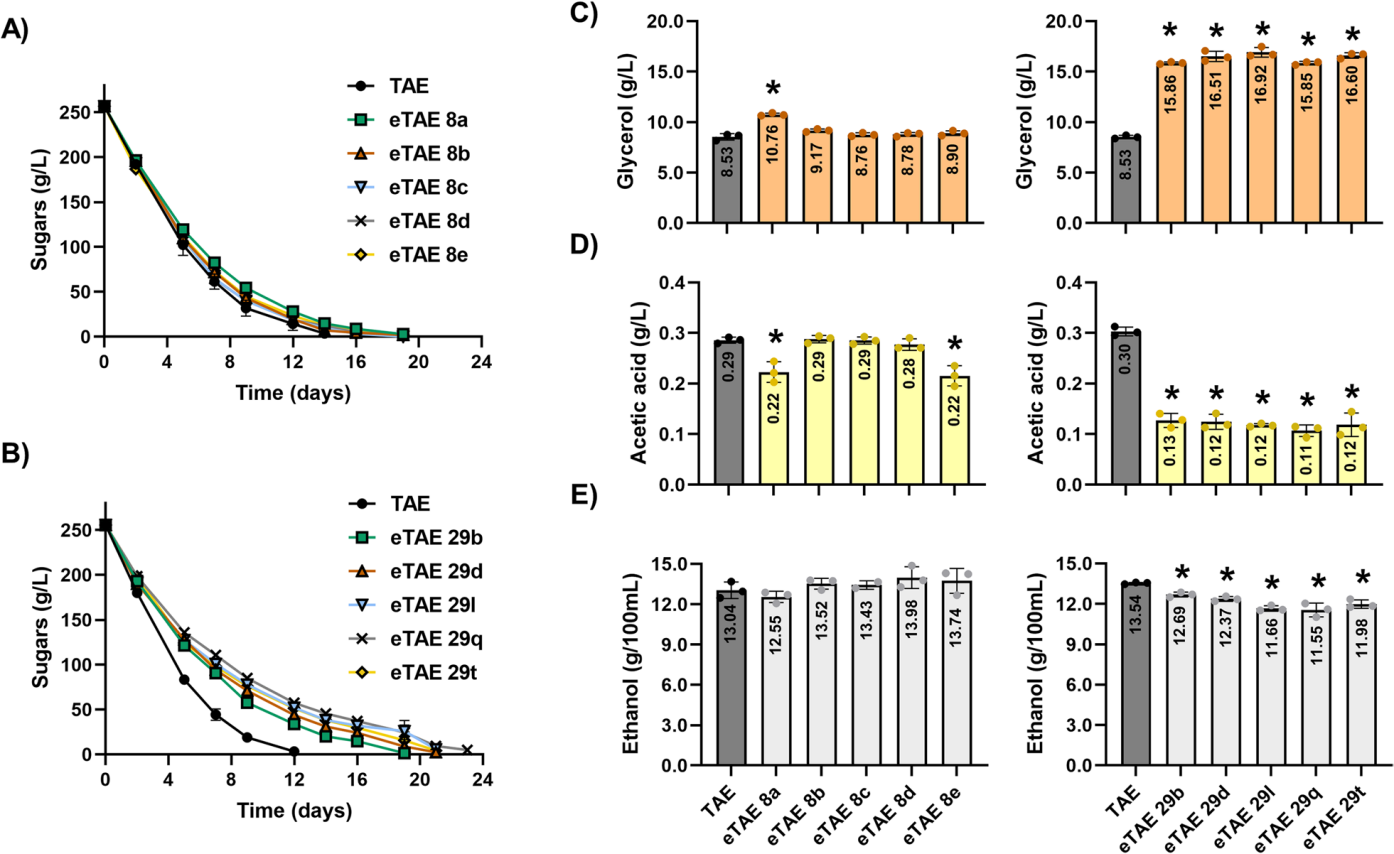
Winemaking performance of five individual clones from the TAE-evolved population in natural red must. **A** Reducing sugar consumption (from the initial concentration of 250 g/L of the natural red grape must, Bobal variety) during fermentation in clones isolated after 8 transfers and **B** after 29 transfers. **C** Glycerol, **D** acetic acid and **E** ethanol produced at the end of fermentation with the individual clones isolated after 8 transfers (left) and 29 transfers (right). Fermentation was carried out in triplicate, and the average and standard deviation are provided. Significant differences (*p < 0.05, Student’s t-test) between the isolated clones and their parental strains are shown. Information regarding the residual sugars at the end of fermentation, as well as the metabolites of oenological interest produced by the clones isolated from the MAE, EAE and TAE evolved populations, where significant differences with respect to the parental strain have been recorded, can be found in Additional file 6

Figure 3 shows the microfermentation experiment with individual clones isolated from the evolution of the TAE strain. Five clones selected after 8 (Fig. 3A) and five after 29 transfers (Fig. 3B) showed a slower fermentation profile than the parental strain, although they completed the consumption of sugars. Metabolites of oenological interest were measured on the last day of fermentation. After 8 transfers, only clone eTAE 8a showed significant differences in final acetic acid and glycerol levels compared to those of the parental strain. The glycerol content of eTAE 8a was 1.26-fold higher than that of the TAE strain (Fig. 3C), and the acetate levels were 0.76-fold lower (Fig. 3D), but no significant differences in ethanol production were detected (Fig. 3E). However, after 29 transfers all the evolved clones showed an increase in glycerol up to 1.98-fold (Fig. 3C), a decrease in acetic acid up to 0.37-fold (Fig. 3D) and up to 0.85-fold decrease in ethanol (Fig. 3E) compared to those of the parental strain.

Regarding the clones isolated from the MAE and EAE ALE experiments, Additional file 6 shows those in which significant differences in acetate, glycerol and/or ethanol were recorded compared to the parental strain. After 8 and 29 transfers of MAE evolution, several clones exhibited reduced acetate levels and increased glycerol production compared to those of the parental strain. However, only clones eMAE 29-2a and eMAE 29-2c (obtained after 29 transfers) showed a significant decrease in ethanol production. From the evolution of EAE, after 29 transfers, only two clones (eEAE 29j and eEAE 29o) were obtained that showed higher glycerol and lower acetate levels than the parental strain, although with no significant differences in ethanol.

### Activation status of the retrograde pathway in evolved mutants

After verifying that the selected evolved mutants showed increased resistance to AEC (Fig. 2c and Additional file 5C) and a similar phenotype to the *MKS1* null mutant in winemaking (more glycerol, less acetate and sometimes less ethanol) ([19], Fig. 3 and Additional file 6), the next step was to study whether they also had a hyperactivated Retrograde pathway.

For this purpose, the evolved mutants with the most differential phenotypes in winemaking were selected. After 8 transfers, eMAE 8-1b, eMAE 8-2d and eTAE 8a were selected because they showed the greatest reduction in acetic acid and increase in glycerol compared to their parental strain. For the same reasons, eEAE 29j and eEAE 29o were selected after 29 transfers, and eMAE 29-2c and eTAE 29 l, as they also showed reduced ethanol production. From exponential growing cells in glucose-rich medium (conditions in which the Retrograde pathway is repressed [28, 32]), quantitative real-time PCR was performed to verify the relative expression levels of two canonical RR marker genes, *CIT2* and *DLD3* [7, 31]. *CIT2* encodes the peroxisomal isoform of citrate synthase, and *DLD3* is a 2-hydroxyglutarate transhydrogenase that produces lactate from pyruvate. Both enzymes promote α-ketoglutarate synthesis in cells with mitochondrial dysfunction [1, 33].

**Fig. 4:**
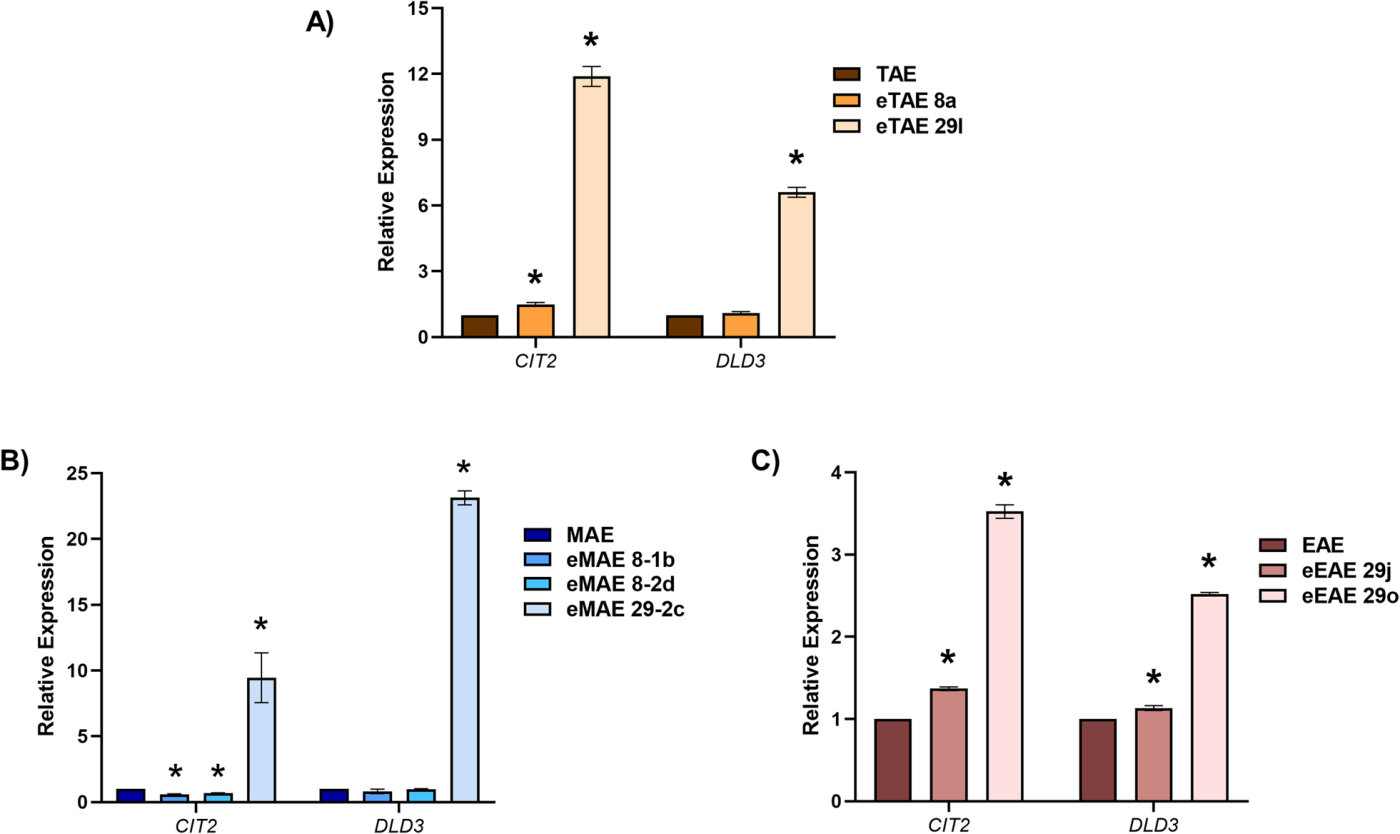
mRNA levels of the Retrograde Response targets *CIT2* and *DLD3* in selected mutants. **A** Relative expression levels in selected clones isolated after 8 and 29 transfers from the evolution of MAE, **B** from the evolution of TAE and **C** from the evolution of EAE. Cells are isolated from an exponential culture in rich medium YPD, where RR targets are repressed. The data are presented as averages of three independent experiments with standard errors. Significant differences (*p < 0.05, Student’s t-test) between the isolated clones and their parental strains are shown

Among the clones selected after 8 transfers for MAE and TAE, only eTAE 8a showed a slightly higher increase in *CIT2* relative expression than did the parental strain, but there was no difference in *DLD3* expression (Fig. 4A). Contrary to our assumption, *CIT2* transcription levels in eMAE 8-1b and eMAE 8-2d were significantly lower than those in MAE (Fig. 4B), so the phenotype of these clones in winemaking was not linked to overactivated Retrograde signalling. However, after 29 transfers, all the selected mutants showed increased expression of *CIT2* and *DLD3*, although to a lesser extent for eEAE 29j (Fig. 4C).

### Whole-Genome sequencing of selected evolved mutants

**Fig. 5:**
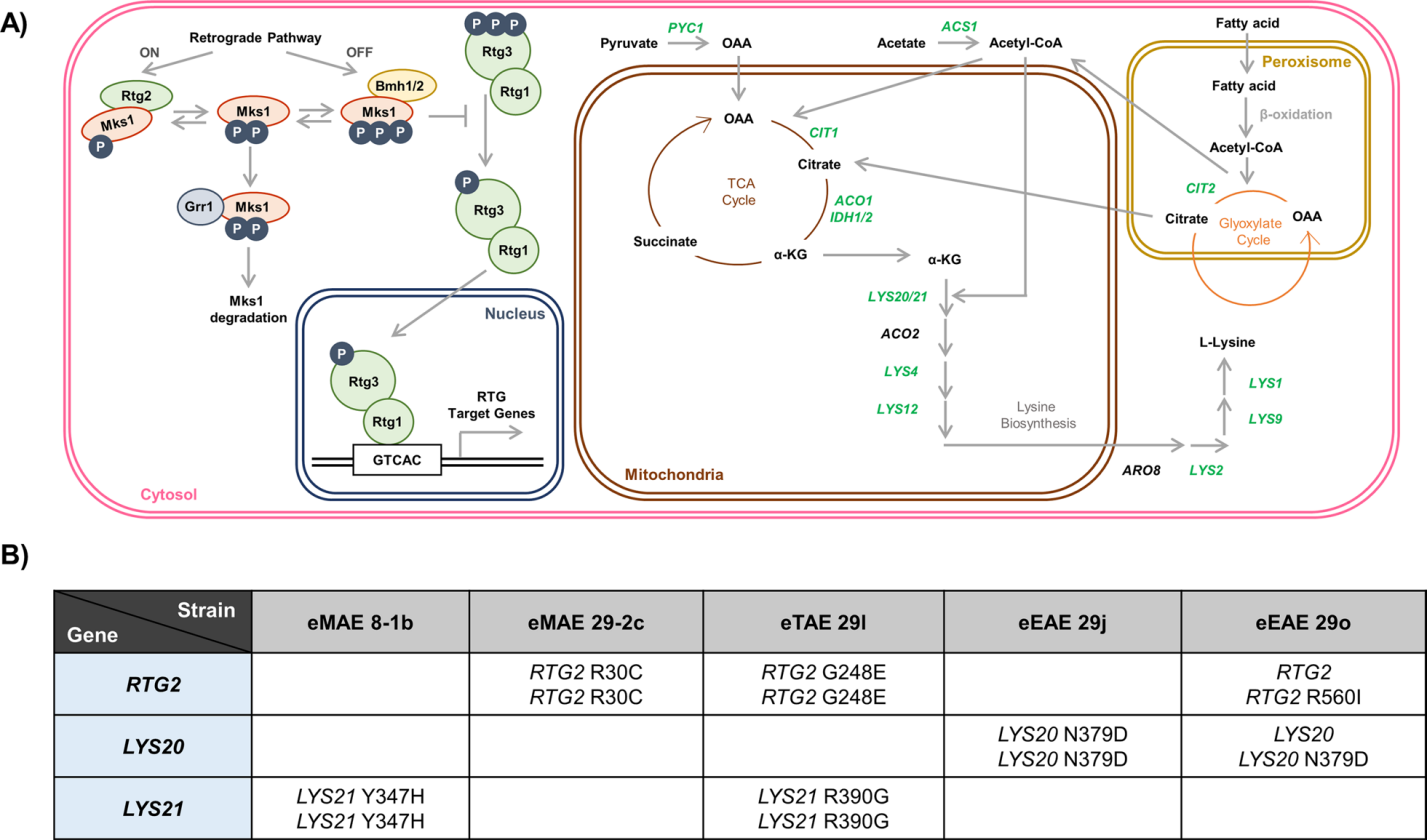
Targeted search for mutations in genes involved in RR-dependent lysine biosynthesis. **A** Summary of the pathways, genes and metabolites involved in lysine biosynthesis. RR-dependent gene expression is based on a dynamic interaction between Rtg2 and Mks1. When the RR pathway is inactive, Mks1p dissociates from Rtg2p and interacts with the 14–3-3 protein Bmh1/2 to inhibit Rtg1/3 nuclear translocation. Grr1-dependent degradation of free Mks1 ensures an efficient switch between the Rtg2-Mks1 and Bmh1/2-Mks1 complexes. The TCA cycle reactions that turn succinate into oxaloacetate are rendered inactive when Retrograde signalling occurs. However, the TCA cycle can be fuelled by citrate generated in the glyoxylate cycle, which requires only a source of acetyl-CoA. The β-oxidation of fatty acids or other sources may provide this acetyl-CoA. The TCA cycle can also be sustained by the anaplerotic conversion of pyruvate to oxaloacetate (OAA) in reactions initiated by pyruvate carboxylase. This over-supplementation of OAA and the connection to the glyoxylate cycle allows the TCA cycle to remain as a net source of α-ketoglutarate (α-KG) for lysine biosynthesis. The condensation of α-KG and acetyl-CoA, catalysed by Lys20/21 homocitrate synthases, is the first step of the lysine biosynthetic pathway in *S. cerevisiae*. According to [12], upregulated genes in the *mks1*∆ deletion mutant are indicated in green. *ACS1*, acetyl-coenzyme A synthetase; *PYC1*, pyruvate carboxylase; *CIT1*, mitochondrial citrate synthase; *ACO1/2*, aconitases; *IDH1/2*, isocitrate dehydrogenases 1 and 2; *CIT2*, peroxisomal citrate synthase; *LYS20/21*, homocitrate synthases; *LYS4*, homoaconitase; *LYS12*, Homo-isocitrate dehydrogenase; *ARO8*, aminotransferase; *LYS2*, α-aminoadipate reductase; *LYS9* and *LYS21*, Saccharopine dehydrogenases. **B** Table of gene mutations found in selected clones from each independently evolved population

To investigate the genetic basis behind the obtained phenotypes, we performed whole-genome sequencing on the mutants with higher RR expression, namely, eMAE 29-2c, eTAE 29 l, eEAE 29j and eEAE 29o. The eMAE 8-1b mutant was also included with the aim of identifying mutations, independent of the Retrograde pathway, causing its interesting phenotype in winemaking. Due to the differences between industrial strains and the reference *S. cerevisiae* genome and because commercial strains are diploid while laboratory strains are used and sequenced as haploid strains, it was difficult to perform a direct comparison. Lists for the unique exonic non-synonymous SNPs and in/del compared to each parental strain, that was sequenced in parallel, were obtained (Additional file 4). A visual analysis ignoring the subtelomeric regions that exhibited the most changes, mainly focusing on the genes involved in the Retrograde pathway and lysine biosynthesis, was performed (Fig. 5A). Figure 5B shows an overview of the relevant genes mutated in the selected evolved strains. As all mutant strains contained mutations compatible with the phenotype observed, the rest of the mutations, which were mostly present in heterozygosis (and therefore are less stable), were excluded. Mutations in *RTG2*, which encodes the RR activator Rtg2, seem of outstanding relevance due to their presence in three of the five sequenced strains. Moreover, the variety of mutations found in this gene is a further indication of its importance in AEC resistance. In strains eMAE 29-2c and eTAE 29 l there were two homozygous mutations resulting in two different amino acid changes, while in strain eEAE 29o there was another different amino acid substitution, but in this case, it was heterozygous.

Mutations in the *LYS20* and *LYS21* genes were also found, as previously described in studies where AEC-resistant mutants were isolated [17, 20]. These genes encode two paralogous homocitrate synthases, Lys20 and Lys21, which catalyse the first step of lysine biosynthesis, using 2-oxoglutarate and acetyl-CoA (Fig. 5A). In strains evolved from EAE, the same mutation was found in *LYS20*, but only in heterozygosis in the eEAE 29o strain. In eMAE 8-1b and eTAE 29 l, different homozygous mutations were found in *LYS21*. In an attempt to isolate any of the two eEAE 29o mutations in homozygosis, the same adaptive evolution experiment with AEC was extended for an additional 29 transfers but without success (data not shown).

### Analysis of isolated gene mutations

**Fig. 6:**
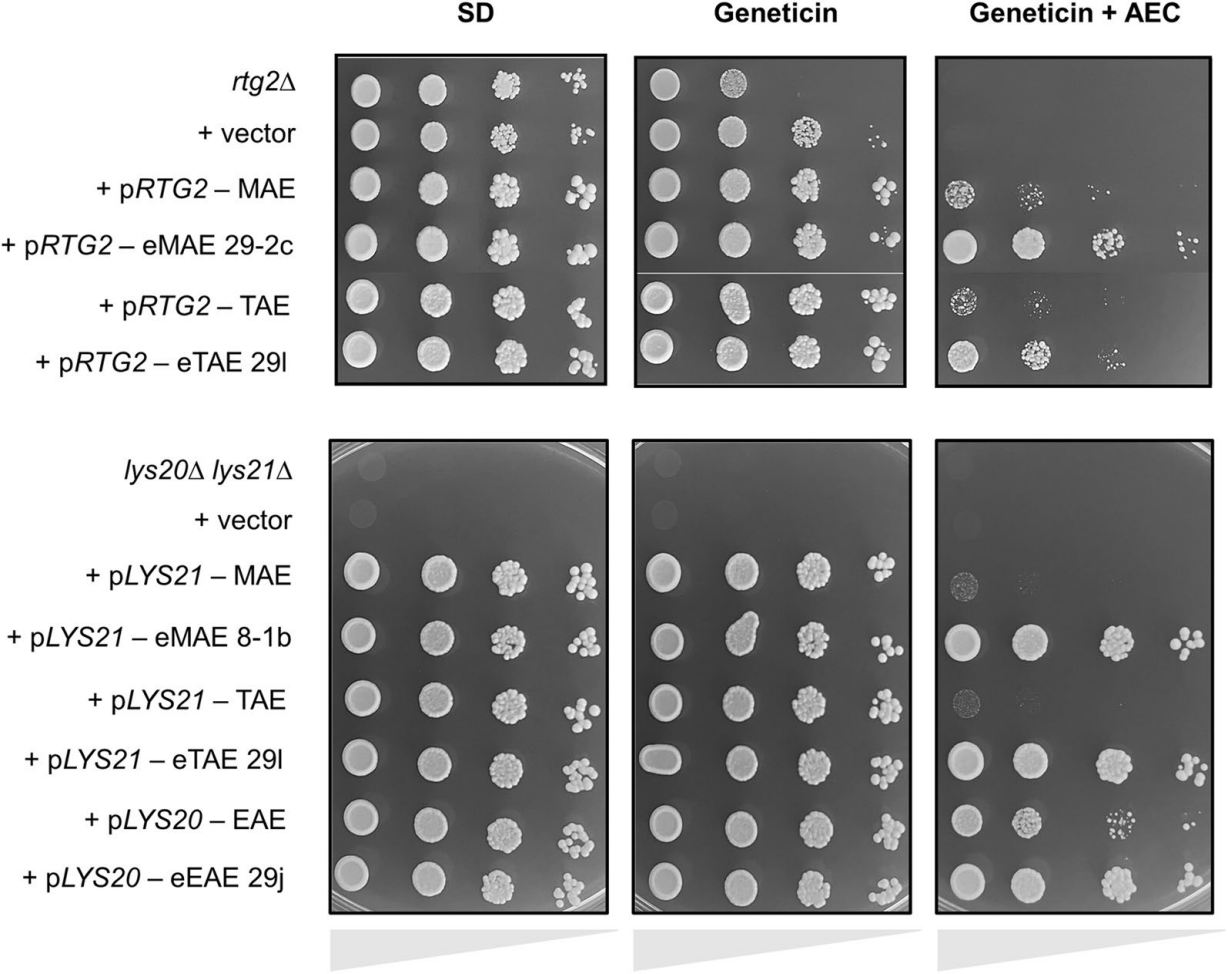
Involvement of mutations identified in the *RTG2*, *LYS20* and *LYS21* genes on the thialysine resistance phenotype. Spot growth analysis of C9 *rtg2∆* and C9 *lys20∆lys21∆* mutants containing the empty vector (+ vector), the *RTG2*, *LYS20* and *LYS21* alleles of the parental strains, or the alleles carrying the mutations identified in eMAE 8-1b, eMAE 29-2c, eTAE 29 l and eEAE 29j evolved strains. A 5-μl volume of each serially diluted culture (from 10^–1^ to 10^–4^) was spotted on SD (untreated) plates or SD plates containing 200 mg/L geneticin with or without 35 mg/L AEC. All plates were incubated at 30 °C for 48 h

In order to find the genetic causes of the observed phenotypes, we selected evolved genes with homozygous mutations (*RTG2* – eMAE 29-2c, *RTG2* – eTAE 29 l, *LYS21* – eMAE 8-1b, *LYS21* – eTAE 29 l, *LYS20* – eEAE 29j) and cloned them into a centromeric plasmid under their own promoter. The resulting constructs were subsequently transformed into the corresponding *rtg2∆* or *lys20∆lys21∆* mutant versions of the haploid wine strain C9 (derived from the commercial strain L2056). The use of a haploid version facilitated the deletion of the genes and introduced another genetic background, which would reinforce the relevance of the mutations. Figure 6 shows a spot analysis test to verify the implication of the isolated mutations in AEC tolerance compared with the parental gene isolated from each genetic background (which in all cases was identical to the reference genome). Mutant alleles of *RTG2* conferred greater resistance to AEC than did the parental alleles, and the effect was most severe in the case of the R30C mutation isolated from eMAE 29-2c. The *lys20∆lys21∆* mutant strain was unable to grow on minimal medium given its inability to synthesize lysine, a defect that was alleviated by introducing at least one of the *LYS20* or *LYS21* versions, confirming their functional overlap. Again, the *LYS20/21* alleles of the evolved strains conferred greater resistance to AEC than did the alleles of the parental strains. This effect is less apparent in *LYS20,* as the presence of the parental strain allele already confers some resistance to the toxic, demonstrating that this homocitrate synthase isoform exerts greater control over lysine synthesis, as described in previous works [43, 44]. Furthermore, the AEC tolerance phenotype conferred by these mutations was observed when the C9 wild-type strain (and the *lys20∆* and *lys21∆* single mutants) was transformed, indicating the dominant nature of such mutations (Additional file 7).

**Fig. 7:**
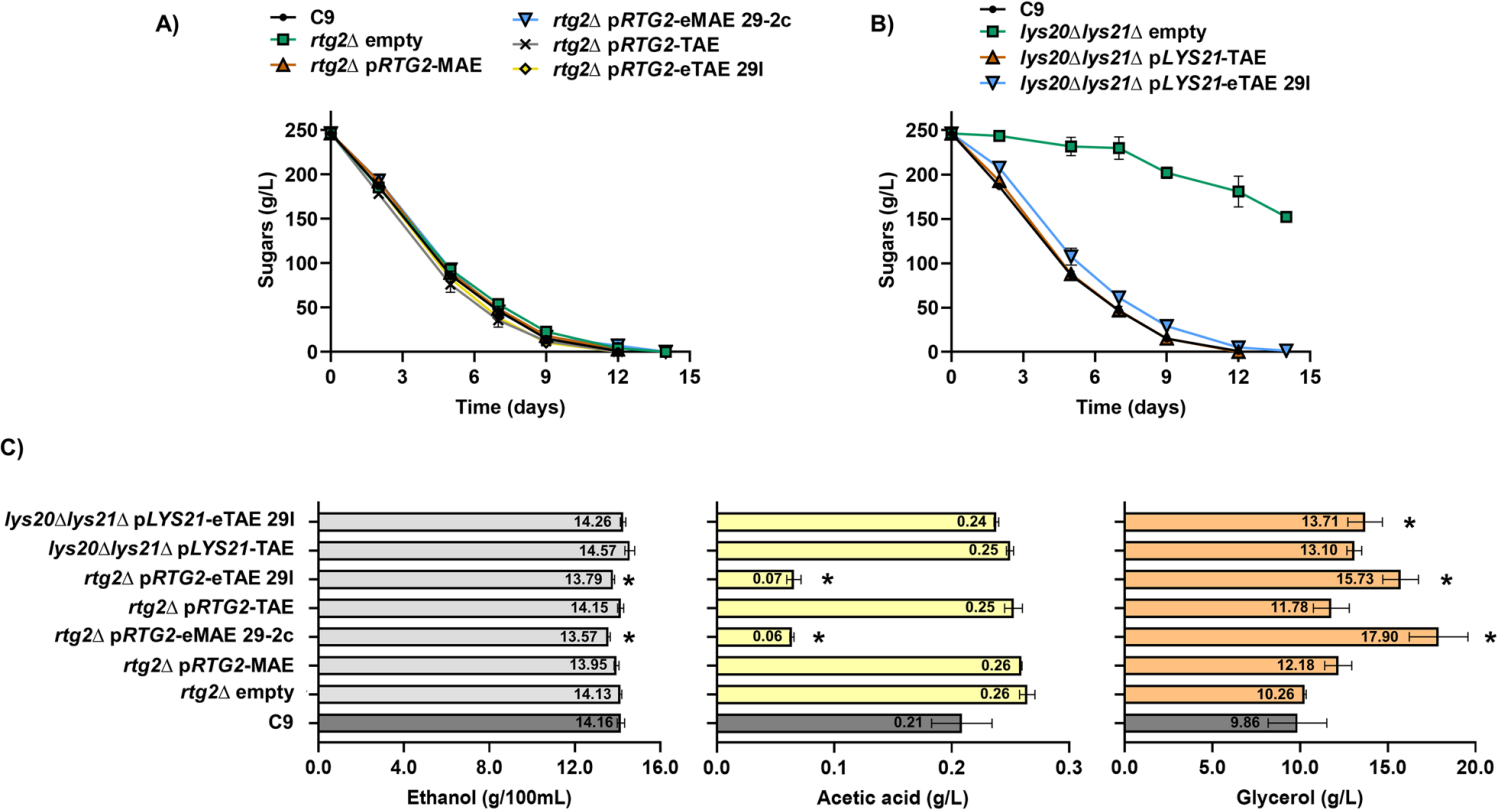
*RTG2* mutations were responsible for increasing glycerol and decreasing acetic acid and ethanol during winemaking. **A** Laboratory-scale wine fermentations in natural grape must (Bobal variety) with an initial concentration of reducing sugars of 250 g/L were carried out with the strain C9, the *rtg2∆* mutant carrying the empty vector or containing different *RTG2* alleles **B** and the *lys20∆lys21∆* mutant carrying the empty plasmid or containing the eTAE 29 l *LYS21* allele were monitored by measuring sugar consumption. **C** Ethanol, acetic acid and glycerol levels produced at the end of fermentation by each strain. The experiments were performed in triplicate, and the means and standard deviations are provided. Significant differences (*p < 0.05, Student’s t-test) between the C9 strain and the mutants containing the plasmids are described in Additional file 2

The next step was to determine whether these mutations were responsible for the phenotype in winemaking. For this purpose, microvinification experiments were carried out on red grape juice, restricted only to those mutations isolated in homozygosis that were also responsible for hyperactivation of the Retrograde Response (Fig. 7). The *lys20∆lys21∆* strain with the empty plasmid (*lys20∆lys21∆* empty) was discarded for further measurements as it was unable to consume the sugars present in the must (Fig. 7B), highlighting the importance of the amino acid lysine under winemaking conditions. The rest of the strains consumed all sugars, although with a slight delay in the case of the *rtg2∆* empty plasmid strain, and the strains carrying the mutation in *RTG2* of eMAE 29-2c (Fig. 7A) and the mutation in *LYS21* of eTAE 29 l (Fig. 7B). Ethanol, acetic acid and glycerol levels were measured at the end of fermentation (Fig. 7C). Compared with the C9 wild-type strain, the *rtg2∆* strain showed no difference in glycerol or acetate levels, and there were no differences in ethanol levels either as described for Tempranillo variety grape juice [41] or in other genetic backgrounds [19]. The *LYS21* mutation isolated from eTAE 29 l resulted in a slight increase in glycerol levels but did not decrease ethanol or volatile acidity. Mutations in *RTG2*, isolated in eTAE 29 l and eMAE 29-2c, led to a reduction in ethanol and acetic acid levels, as well as an increase in glycerol content. Therefore, mutations in *RTG2* were responsible for the winemaking phenotype of the evolved strains, while the *LYS21* mutation from eTAE 29 l by itself did confer resistance to AEC but was not able to reduce volatile acidity or ethanol.

### Pilot-scale wine fermentation in experimental cellar

**Fig. 8:**
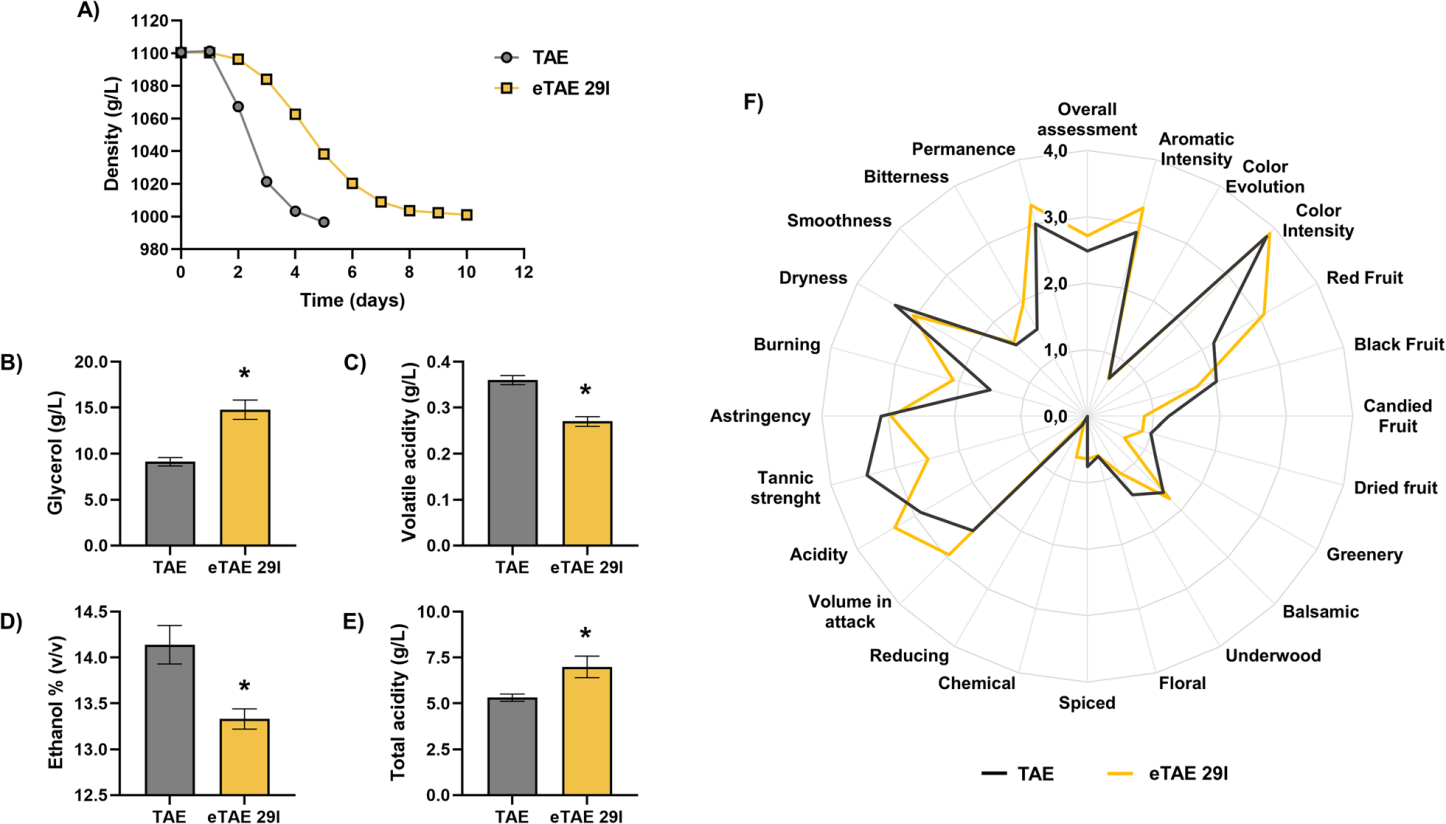
Exploring the suitability of the eTAE 29 l evolved strain in pilot-scale fermentations. **A** The progress of alcoholic fermentation by the evolved strain eTAE 29 l and its parent strain TAE was monitored by density control. **B** Glycerol, **C** acetic acid (expressed as volatile acidity), **D** ethanol and **E** total acidity were measured at the end of fermentation. **F** Sensory description of the wine samples**.** Fermentation was carried out in triplicate, and the average and standard deviations are provided. Significant differences (* p < 0.05, Student’s t-test) are shown

Since strain eTAE 29 l hyperactivated Retrograde signalling and showed the greatest ethanol reduction (as well as increased glycerol and reduced acetic acid levels) (Fig. 3 and Additional file 6), it was selected for evaluation of its potential for use under industrial conditions. Wine fermentations were carried out in an experimental cellar with 50 kg of red grapes (variety Tempranillo) to determine whether the results at a larger scale were consistent with those observed at the laboratory scale. Figure 8 shows the results obtained from the pilot-scale fermentations. As expected, the fermentation of eTAE 29 l was slower than that of its parental strain TAE (Fig. 8A). However, eTAE 29 l produced significantly more glycerol (Fig. 8B) and less acetic acid (volatile acidity) (Fig. 8C), as described in the microfermentation experiments. In addition, it also achieved a significant reduction of 0.8% (v/v) in ethanol (Fig. 8D). In addition, it increased the total acidity, measured analytically as tartaric acid (Fig. 8E), but did not affect the levels of other acids, such as L-malic acid, or significantly reduced the final pH (data not shown). Finally, an organoleptic analysis was performed to rule out the possibility that the mutation produced any unpleasant secondary effects. Sensory analysis of the wines obtained after fermentation (Fig. 8F) revealed that eTAE 29 l contributed a higher presence of red fruit, but less greenery and black, candied and dried fruit. In addition, the wines resulting from fermentation with eTAE 29 l were judged to have greater volume on attack, aromatic intensity, acidity and less dryness, although they had less tannic strength and a slight chemical touch. Despite the increase in glycerol levels by this strain, it did not affect the unctuousness of the wine. Overall, the aromatic intensity and global score were best for the evolved strain, so the mutations did not cause any undesired effects on the final product.

## Discussion

Global warming has various effects on the wine industry, including an increase in alcohol content. The framework of the present research is based on the idea of reducing ethanol levels during wine fermentation by improving yeasts, avoiding the drawbacks of excess volatile acidity and increasing glycerol production. To address these challenges, in recent years, there has been a growing trend to explore the potential of non-*Saccharomyces* yeasts [27, 58]. However, the use of non-conventional yeasts requires additional inoculation strategies [5], as well as optimization of their production [51] and nutrient management [36], among other parameters. Therefore, direct breeding of *S. cerevisiae* wine strains is currently more straightforward, given its industrial know-how and the simplicity of the process.

The research of our laboratory has been focused on *S. cerevisiae* nutrient signalling pathways as a target for improvement. In line with our previous works, genetic engineering was used for generating mutants at key points in these pathways that, despite not being suitable for the market, can provide valuable phenotypic information that can be used later for targeted development of new strains [19, 52, 54]. Alternatively to genetic manipulation, chemical inhibitors of such pathways can also be used to test hypotheses and improve phenotypes [53, 55] or to generate new ones through directed evolution strategies [23]. Recently, we reported that hyperactivation of the Retrograde Response pathway, by deletion of its repressor *MKS1*, changes carbon metabolism by increasing glycerol and reducing ethanol during winemaking [19].

In this work, we developed an ALE-driven approach (Fig. 1) for obtaining non-recombinant *S. cerevisiae* commercial wine strains that reduce ethanol and acetic acid levels in winemaking, without giving up the beneficial characteristics provided by glycerol, as described for the *mks1*∆ mutant. By using this strategy, the resulting strains can be applied at the industrial level, as they are non-GMOs and therefore avoid regulatory constraints and poor consumer acceptance [60]. To this end, yeast cultures of three different commercial wine strains were serially transferred under the pressure of a constant concentration of the toxic lysine analogue AEC. This would lead to an increase in lysine synthesis, ideally as a result of hyperactivation of Retrograde signalling. In *S. cerevisiae*, the lysine biosynthesis pathway starts from α-ketoglutarate, and the production of this intermediate is increased in response to Retrograde stimulation. Therefore, *MKS1* was first known as *LYS80*, and its deletion increases tolerance to this poisonous lysine analogue [16].

After exploring the genomes of selected evolved strains with the expected phenotype, point mutations in genes involved in lysine biosynthesis (*LYS20* and *LYS21*) and/or Retrograde Response (*RTG2*) pathways were detected (Fig. 5B). In most cases, homozygous mutations were detected in these diploid strains. Therefore, we confirmed the AEC suitability to exert selective pressure at specific points along these pathways, as regardless of the experiment and genetic background, all commercial wine strains used showed similar patterns of evolutionary dynamics. *LYS20* and *LYS21* encode two paralogous homocitrate synthases, which catalyse the aldol condensation of α-ketoglutarate and acetyl-CoA to form homocitrate, the first step of the α-amino adipate pathway for lysine biosynthesis (Fig. 5A). This enzymatic reaction is a rate-limiting step because the end-product, lysine, regulates the activity of homocitrate synthases via feedback inhibition [17]. The rational design of these homocitrate synthases has been described as a strategy for constructing new yeast strains with increased lysine productivity [25]. Hence, mutations in these genes could lead to a hyperactivated or retroinhibition-insensitive enzyme that diverts metabolic flux from pyruvate to lysine production, reducing acetyl-CoA and thus acetic acid. This could be the case for the eMAE 8-2d and eEAE 29j strains, with mutations in *LYS21* and *LYS20,* respectively, which show a reduction in acetic acid levels (Additional file 6) without activating the Retrograde pathway (Fig. 4). As they are dominant mutations, they may cause defects in feedback inhibition, but further work is needed to test this hypothesis. Mutations in Ser385 of the *LYS20* gene lead to extreme desensitization to feedback inhibition [25]. Our mutations are located close to that position, for instance, in Asn379 of *LYS20* in eEAE 29j and eEAE 29o, which also points to a similar behaviour.

Surprisingly, all other strains showed mutations in *RTG2* and none were found in the most obvious target, the *MKS1* gene. Rtg2 is a cytoplasmic protein with an N-terminal ATP-binding domain that acts as a sensor of mitochondrial dysfunction and interacts directly with Mks1, allowing the translocation of Rtg1/3 transcription factors from the cytoplasm to the nucleus to activate the expression of RR target genes [18, 34](Fig. 5A). Thus, mutations in *RTG2* could prevent or decrease the repression of Retrograde signalling equivalently to *MKS1* deletion, that may be more pleiotropic, affecting other processes during the evolution experiment. Indeed, the eMAE 29-2c, eTAE 29 l and eEAE 29o strains showed hyperactivation of the *CIT2* and *DLD3* genes, which are subject to Retrograde regulation. *CIT2* encodes the peroxisomal citrate synthase, a glyoxylate cycle enzyme that enables yeast to use two carbon compounds as its only carbon source. In respiration-deficient cells, *CIT2* is overexpressed to increase citrate production from acetyl-CoA and oxaloacetate, providing the metabolic intermediates necessary for anabolic lysine or glutamate biosynthesis from α-ketoglutarate [33]. *DLD3*, encoding a potential cytoplasmic isoform of D-lactate dehydrogenase, is also overexpressed in cells with dysfunctional mitochondria [7]. It has been described that Dld3 in fact acts as a FAD-dependent transhydrogenase that uses pyruvate as a hydrogen acceptor to convert D-2-hydroxyglutarate to α-ketoglutarate [1]. Therefore, during vinification, these strains could undergo an increase in glycerol levels at the expense of glycolytic flux, reducing ethanol without increasing volatile acidity, as pyruvate is being pushed towards α-ketoglutarate synthesis, consuming acetyl-CoA in the process (Fig. 3**, **Additional file 6). Another explanation could be that, in these evolved strains, the ethanol produced during wine fermentation is taken up to supply most of the cytosolic and mitochondrial acetyl-CoA [61]. Further studies using omics approaches are needed to elucidate the metabolic fluxes of these mutants during grape must fermentation.

Regardless of the mutations accumulated by the evolved strains, all of them were more resistant to AEC than their respective ancestral strains (Fig. 2, Additional file 5C). However, for laboratory-scale vinifications, after 8 transfers of ALE, most of these strains did not give a final product with the desired characteristics (Fig. 3, Additional file 6). It is expected that mutations are first produced in one of the alleles of these diploid strains and the mutation may be fixed in the other copy by gene conversion, due to the high homologous recombination activity of yeast. Alternatively, this may be because the selective pressure exerted by the AEC first forces mutations at the level of the lysine synthesis pathway and later at the Retrograde pathway. Our study shows that this can stabilize the mutations by extending the evolution experiment without the need to increase the concentration of the toxic agent. By studying the effect of the mutations described in the sequenced evolved strains, we observed that these mutations were indeed responsible for the AEC resistance phenotype in these strains (Fig. 6). Moreover, the effect of these alleles dominates over those of the wild-type strain (Additional file 7). As described, the Lys20 homocitrate synthase isoform exerts greater control over lysine synthesis [43, 44]. Therefore, it is possible that the N379D mutation in one of the *LYS20* copies of strain eEAE 29o was potent enough to decrease the AEC selective pressure. This would explain why eEAE 29o showed mutations in *LYS20* and *RTG2* in heterozygosis, even prolonging its evolution for another 29 transfers. This time, it would have been interesting to increase the AEC concentration during the evolution of this strain to exert greater selective pressure and fix the mutations in homozygosity.

Pilot-scale trials on Tempranillo grape must with the eTAE 29 l strain were in line with those carried out in the laboratory, achieving a 0.8% (v/v) reduction in ethanol. The wine produced was evaluated by a panel of experts and was assessed positively without detecting any significant defects (Fig. 8). During ALE experiments, yeast is under almost invariant conditions, so it is not uncommon to observe that evolved strains show lower performance or a different phenotype when returned to an industrially relevant environment. This may be due to genetic drift or offsets of genetic modifications [15]. Therefore, it was crucial to scale up the winemaking process and confirm that the overall good attributes of the eTAE 29 l strain obtained in this study were maintained.

Taken together, our study demonstrated that through adaptive evolution, it is possible to obtain non-genetically modified industrial *S. cerevisiae* wine strains with hyperactivated Retrograde signalling by accumulating mutations in *RTG2*, the activator of this pathway. These evolved yeasts, by showing a diversification of carbon metabolism, give rise to low-alcohol wines with reduced volatile acidity but high glycerol content. This metabolic redistribution may lead to specific adverse effects, such as a decrease in the fermentation rate, which the industry should consider for its application. However, no defect in growth was detected (see the control plates on Fig. 2C). To completely assess the utility of these strains for the wine industry, more pilot-scale studies on various grape must types and winemaking conditions, together with a sensory examination of the wines developed, are needed.

## Conclusions

Understanding the functioning of nutrient signalling pathways in *S. cerevisiae* wine strains has the potential to aid in the development of new and improved strains resulting in a wine with better characteristics. Our study showed that adaptive evolution in the presence of AEC is a promising strategy for obtaining non-recombinant, low-ethanol-producing yeasts by increasing Retrograde signalling. Evolved mutants with an overactive Retrograde Response led to a reduction of volatile acidity in wine, as they allocate carbon skeletons to lysine production (from ketoglutarate) to acquire tolerance to its toxic analogue. In addition, these mutants overproduce glycerol at the expense of glycolytic flux, so they are also able to reduce ethanol during the fermentation of grape must. One mutant tested in an experimental cellar showed that yeast performance was improved without impairing the organoleptic characteristics of the final product.

### Supplementary Information

Additional file 1: List of the strains used in this study.

Additional file 2: Plasmids used in this work.

Additional file 3: Primers used in this work

Additional file 4: Statistics of mapping, accession numbers, and unique SNPs and InDels for the five evolved strains. Relevant mutations in *RTG2* and *LYS20/21* are indicated in bold.

Additional file 5: Monitoring adaptive laboratory evolution experiments of the TAE and EAE strains and thialysine-resistance characterization of all individual clones isolated after 8 and 29 transfers from the MAE, TAE and EAE evolved populations. (A) Generations obtained in each of the transfers performed during the directed evolution of the TAE and EAE strains and (B) accumulated generations throughout the experiment. The black arrows indicate 8 and 29 transfers, the time points at which individual clones were isolated from the evolved population. (C) Spot growth analysis to test the tolerance of each clone isolated from MAE, TAE and EAE evolutions after 8 and 29 transfers.

Additional file 6: Values of the main metabolites and residual sugars at the end of fermentation. Glycerol, acetic acid and ethanol levels of the isolated evolved clones after 8 and 29 transfers, that registered significative differences compared to the parental strain at the end of fermentations in sterilised natural red grape must (Bobal variety) with an initial concentration of reducing sugars of 250 g/L. The final values for residual sugars have also been indicated. Fermentation was carried out in triplicate, and the average and standard deviations are provided. Statistical differences (* p < 0.05, Student’s t-test) between the evolved clones and their parental strains are shown.

Additional file 7: Involvement of mutations identified in the *RTG2*, *LYS20* and *LYS21* genes on the thialysine resistance phenotype. Spot growth analysis of C9, C9 *lys20∆* and C9* lys21∆* mutants containing the empty vector (+ vector), the *RTG2*, *LYS20* and *LYS21* alleles of the parental strains, or the alleles carrying the mutations identified in eMAE 8-1b, eMAE 29-2c, eTAE 29l and eEAE 29j evolved strains. A 5-μl volume of each serially diluted culture (from 10^-1^ to 10^-4^) was spotted onto SD (untreated) plates or SD plates containing 200 mg/L geneticin with or without 35 mg/L AEC. All plates were incubated at 30°C for 48h.

## Data Availability

Data is provided within the manuscript or supplementary information files. Sequence data have been deposited in NCBI under accession codes PRJNA1105211, PRJNA1105213, PRJNA1105003, PRJNA1105208 and PRJNA1105207.
