# Supplementary material for: Activation of the yeast Retrograde Response pathway by adaptive laboratory evolution with S-(2-aminoethyl)-L-cysteine reduces ethanol and increases glycerol during winemaking
Source: Microb Cell Fact. 2024 Aug 20;23:231. doi: 10.1186/s12934-024-02504-z (PMC11337681; doi:10.1186/s12934-024-02504-z)
Supplement: Supplementary file 1 — Additional file 1: List of the strains used in this study. [file 12934_2024_2504_MOESM1_ESM.pdf]

Additional file 1. List of the strains used in this study.

Yeast Strain	Genotype / Plasmid added	Source
MAE	MAE	Lallemand
MAE *mks1Δ*	MAE CRISPR/Cas9 *mks1Δ*	Garrigós et al., 2023
eMAE 8-1a	MAE evolved in SD+AEC after 8 transfers, culture 1 colony a	This study
eMAE 8-1b	MAE evolved in SD+AEC after 8 transfers, culture 1 colony b	This study
eMAE 8-1c	MAE evolved in SD+AEC after 8 transfers, culture 1 colony c	This study
eMAE 8-1d	MAE evolved in SD+AEC after 8 transfers, culture 1 colony d	This study
eMAE 8-1e	MAE evolved in SD+AEC after 8 transfers, culture 1 colony e	This study
eMAE 8-2a	MAE evolved in SD+AEC after 8 transfers, culture 2 colony a	This study
eMAE 8-2b	MAE evolved in SD+AEC after 8 transfers, culture 2 colony b	This study
eMAE 8-2c	MAE evolved in SD+AEC after 8 transfers, culture 2 colony c	This study
eMAE 8-2d	MAE evolved in SD+AEC after 8 transfers, culture 2 colony d	This study
eMAE 8-2e	MAE evolved in SD+AEC after 8 transfers, culture 2 colony e	This study
eMAE 29-1a	MAE evolved in SD+AEC after 29 transfers, culture 1 colony a	This study
eMAE 29-1b	MAE evolved in SD+AEC after 29 transfers, culture 1 colony b	This study
eMAE 29-1c	MAE evolved in SD+AEC after 29 transfers, culture 1 colony c	This study
eMAE 29-1d	MAE evolved in SD+AEC after 29 transfers, culture 1 colony d	This study
eMAE 29-1e	MAE evolved in SD+AEC after 29 transfers, culture 1 colony e	This study
eMAE 29-2a	MAE evolved in SD+AEC after 29 transfers, culture 2 colony a	This study
eMAE 29-2b	MAE evolved in SD+AEC after 29 transfers, culture 2 colony b	This study
eMAE 29-2c	MAE evolved in SD+AEC after 29 transfers, culture 2 colony c	This study
eMAE 29-2d	MAE evolved in SD+AEC after 29 transfers, culture 2 colony d	This study
eMAE 29-2e	MAE evolved in SD+AEC after 29 transfers, culture 2 colony e	This study
TAE	TAE	Lallemand

TAE *mks1∆*	TAE CRISPR/Cas9 *mks1∆*	Garrigós et al., 2023
eTAE 8a	TAE evolved in SD+AEC after 8 transfers, colony a	This study
eTAE 8b	TAE evolved in SD+AEC after 8 transfers, colony b	This study
eTAE 8c	TAE evolved in SD+AEC after 8 transfers, colony c	This study
eTAE 8d	TAE evolved in SD+AEC after 8 transfers, colony d	This study
eTAE 8e	TAE evolved in SD+AEC after 8 transfers, colony e	This study
eTAE 29b	TAE evolved in SD+AEC after 29 transfers, colony b	This study
eTAE 29d	TAE evolved in SD+AEC after 29 transfers, colony d	This study
eTAE 29l	TAE evolved in SD+AEC after 29 transfers, colony l	This study
eTAE 29q	TAE evolved in SD+AEC after 29 transfers, colony q	This study
eTAE 29t	TAE evolved in SD+AEC after 29 transfers, colony t	This study
EAE	EAE	Lallemand
EAE *mks1∆*	EAE CRISPR/Cas9 *mks1∆*	Garrigós et al., 2023
eEAE 8a	EAE evolved in SD+AEC after 8 transfers, colony a	This study
eEAE 8b	EAE evolved in SD+AEC after 8 transfers, colony b	This study
eEAE 8c	EAE evolved in SD+AEC after 8 transfers, colony c	This study
eEAE 8d	EAE evolved in SD+AEC after 8 transfers, colony d	This study
eEAE 8e	EAE evolved in SD+AEC after 8 transfers, colony e	This study
eEAE 29j	EAE evolved in SD+AEC after 29 transfers, colony j	This study
eEAE 29k	EAE evolved in SD+AEC after 29 transfers, colony k	This study
eEAE 29n	EAE evolved in SD+AEC after 8 transfers, colony n	This study
eEAE 29o	EAE evolved in SD+AEC after 29 transfers, colony o	This study
eEAE 29s	EAE evolved in SD+AEC after 29 transfers, colony s	This study
C9	C9 Mat a *ho*::loxP	Walker et al., 2003
C9 *lys20∆*	C9 *lys20*::loxP	This study

C9 *lys21*Δ	C9 *lys21*::loxP	This study
C9 *lys20*Δ *lys21*Δ	C9 *lys20*::loxP *lys21*::loxP	This study
C9 *rtg2*Δ	C9 *rtg2*::loxP	This study
C9 vector	C9 with plasmid pCUP1pNuiHA kanMX CEN	This study
C9 p*LYS21* – MAE	C9 with plasmid pLYS21 – MAE	This study
C9 p*LYS21* – eMAE 8-1b	C9 with plasmid pLYS21 – eMAE 8-1b	This study
C9 p*LYS21* – TAE	C9 with plasmid pLYS21 – TAE	This study
C9 p*LYS21* – eTAE 29l	C9 with plasmid pLYS21 – eTAE 29l	This study
C9 p*LYS20* – EAE	C9 with plasmid pLYS20 – EAE	This study
C9 p*LYS20* – eEAE 29j	C9 with plasmid pLYS21 – eEAE 29j	This study
C9 p*RTG2* – MAE	C9 with plasmid p*RTG2* – MAE	This study
C9 p*RTG2* – eMAE 29-2c	C9 with plasmid p*RTG2* – eMAE 29-2c	This study
C9 p*RTG2* – TAE	C9 with plasmid p*RTG2* – TAE	This study
C9 p*RTG2* – eTAE 29l	C9 with plasmid p*RTG2* – eTAE 29l	This study
C9 *lys20*Δ vector	C9 *lys20*Δ with plasmid pCUP1pNuiHA kanMX CEN	This study
C9 *lys20*Δ p*LYS20* – EAE	C9 *lys20*Δ with plasmid p*LYS20* – EAE	This study
C9 *lys20*Δ p*LYS20* – eEAE 29j	C9 *lys20*Δ with plasmid p*LYS20* – eEAE 29j	This study
C9 *lys21*Δ vector	C9 *lys21*Δ with plasmid pCUP1pNuiHA kanMX CEN	This study
C9 *lys21*Δ p*LYS21* – MAE	C9 *lys21*Δ with plasmid p*LYS21* – MAE	This study
C9 *lys21*Δ p*LYS21* – eMAE 8-1b	C9 *lys21*Δ with plasmid p*LYS21* – eMAE 8-1b	This study
C9 *lys21*Δ p*LYS21* – TAE	C9 *lys21*Δ with plasmid p*LYS21* – TAE	This study
C9 *lys21*Δ p*LYS21* – eTAE 29l	C9 *lys21*Δ with plasmid p*LYS21* – eTAE 29l	This study
C9 *lys20*Δ *lys21*Δ vector	C9 *lys20*Δ *lys21*Δ with plasmid pCUP1pNuiHA kanMX CEN	This study

C9 *lys20*Δ *lys21*Δ p*LYS20* – EAE	C9 *lys20*Δ *lys21*Δ with plasmid p*LYS20* – EAE	This study
C9 *lys20*Δ *lys21*Δ p*LYS20* – eEAE 29j	C9 *lys20*Δ *lys21*Δ with plasmid p*LYS20* – eEAE 29j	This study
C9 *lys20*Δ *lys21*Δ p*LYS21* – MAE	C9 *lys20*Δ *lys21*Δ with plasmid p*LYS21* – MAE	This study
C9 *lys20*Δ *lys21*Δ p*LYS21* – eMAE 8-1b	C9 *lys20*Δ *lys21*Δ with plasmid p*LYS21* – eMAE 8-1b	This study
C9 *lys20*Δ *lys21*Δ p*LYS21* – TAE	C9 *lys20*Δ *lys21*Δ with plasmid p*LYS21* – TAE	This study
C9 *lys20*Δ *lys21*Δ p*LYS21* – eTAE 29l	C9 *lys20*Δ *lys21*Δ with plasmid p*LYS21* – eTAE 29l	This study
C9 *rtg2*Δ vector	C9 *rtg2*Δ with plasmid pCUP1pNuiHA kanMX CEN	This study
C9 *rtg2*Δ p*RTG2* – MAE	C9 *rtg2*Δ with plasmid p*RTG2* – MAE	This study
C9 *rtg2*Δ p*RTG2* – eMAE 29-2c	C9 *rtg2*Δ with plasmid p*RTG2* – eMAE 29-2c	This study
C9 *rtg2*Δ p*RTG2* – TAE	C9 *rtg2*Δ with plasmid p*RTG2* – TAE	This study
C9 *rtg2*Δ p*RTG2* – eTAE 29l	C9 *rtg2*Δ with plasmid p*RTG2* – eTAE 29l	This study
