# Supplementary material for: Activation of the yeast Retrograde Response pathway by adaptive laboratory evolution with S-(2-aminoethyl)-L-cysteine reduces ethanol and increases glycerol during winemaking
Source: Microb Cell Fact. 2024 Aug 20;23:231. doi: 10.1186/s12934-024-02504-z (PMC11337681; doi:10.1186/s12934-024-02504-z)
Supplement: Supplementary file 2 — Additional file 2: Plasmids used in this work. [file 12934_2024_2504_MOESM2_ESM.pdf]

Additional file 2. Plasmids used in this work.

Plasmid	Description	Source
pUG6	Multi-copy plasmid (loxP–kanMX–loxP disruption module, kanMX marker)	Güldener et al., 1996
pYEP-cre-cyh	Multi-copy plasmid (GAL1-cre recombinase system, CYH marker)	Delneri et al., 2000
pCUP1pNuiHA kanMX CEN	Centromeric ARS plasmid (kanMX marker, ScCUP1 promoter)	Gift from Nils Johnsson (Addgene plasmid #131168)
p*LYS21* – MAE	pCUP1pNuiHA kanMX CEN with *LYS21* gene from MAE parental strain	This study
p*LYS21* – eMAE 8-1b	pCUP1pNuiHA kanMX CEN with $LYS21^{Y347H}$ gene from eMAE 8-1b evolved strain	This study
p*LYS21* – TAE	pCUP1pNuiHA kanMX CEN with *LYS21* gene from TAE parental strain	This study
p*LYS21* – eTAE 29l	pCUP1pNuiHA kanMX CEN with $LYS21^{R390G}$ gene from eTAE 29l evolved strain	This study
p*LYS20* – EAE	pCUP1pNuiHA kanMX CEN with *LYS20* gene from EAE parental strain	This study
p*LYS20* – eEAE 29j	pCUP1pNuiHA kanMX CEN with $LYS20^{N379D}$ gene from eEAE 29j evolved strain	This study
p*RTG2* – MAE	pCUP1pNuiHA kanMX CEN with *RTG2* gene from MAE parental strain	This study
p*RTG2* – eMAE 29-2c	pCUP1pNuiHA kanMX CEN with $RTG2^{R30C}$ gene from eMAE 29-2c evolved strain	This study
p*RTG2* – TAE	pCUP1pNuiHA kanMX CEN with *RTG2* gene from TAE parental strain	This study
p*RTG2* – eTAE 29l	pCUP1pNuiHA kanMX CEN with $RTG2^{G248E}$ gene from eTAE 29l evolved strain	This study
