# Supplementary material for: Activation of the yeast Retrograde Response pathway by adaptive laboratory evolution with S-(2-aminoethyl)-L-cysteine reduces ethanol and increases glycerol during winemaking
Source: Microb Cell Fact. 2024 Aug 20;23:231. doi: 10.1186/s12934-024-02504-z (PMC11337681; doi:10.1186/s12934-024-02504-z)
Supplement: Supplementary file 3 — Additional file 3: Primers used in this work [file 12934_2024_2504_MOESM3_ESM.pdf]

Additional file 3. Primers used in this work.

Primer	Sequence (5' – 3')	Source and Use
Interdelta analysis		
Delta-12	TCAACAATGGAATCCCAAC	(Legras and Karst, 2003)
Delta-21	CATCTTAACACCGTATATGA	(Legras and Karst, 2003)
Gene deletions		
*LYS20*a	ATAACAAACAAAGGAAGGACCCTGTATTGTTTT CCTAAAGTTCGTACGCTGCAGGTCGAC	This study
*LYS20*b	TGTAATAAGTGAAAGAACATAATGTAATGGTTT GGGAACGATAGGCCACTAGTGGATCTG	" "
*LYS20*c	TTTTAAGGGCCTCATCGCTG	" "
*LYS20*d	TTAGAAAGATAGTCGCCCGG	" "
LYS20e	GCATCACGGGAGAGTAAATC	" "
*LYS21*a	CTTCTTGTTGCGTTTAGGCCTTTATTTCGTACA CATTTAATTCGTACGCTGCAGGTCGAC	" "
*LYS21*b	CGGTAATCGCCAGTACTTAAACACCTAATAACG CCCAGTAATAGGCCACTAGTGGATCTG	" "
*LYS21*c	CTTCTTGTTGCGTTTAGGCC	" "
*LYS21*d	GTATCGAAGAATGCGTTGGC	" "
*LYS21*e	ATAATAGCGTGGGGAATCGC	" "
*RTG2*a	ATGTCAACACTTAGCGATAGTGATACC	" "
*RTG2*b	TCACATACATAATCGTACCAATATACT	" "
*RTG2*c	AGTCACATGACCGCGATAAG	" "
*RTG2*d	TTCTTGCATGATGTGCAGCC	" "
*RTG2*e	TAAACTGGTCTCCACCTCAC	" "
K2	GGGACAATTCAACGCGTCTG	
K3	CCTCGACATCATCTGCCC	
Cloning		
LYS20-X	TAAGCACTCGAGATTTGGTATACTGCGTGCGC	This study
LYS20-B	TGCTTAGGATCCTGAGCGTGCTAATGCTGAAC	" "
*LYS20*p	TCTCGGTTGAGTAAACGCTC	" "
*LYS20*t	TTGCTATCCCAAAGGAGTGC	" "
LYS21-X	TAAGCACTCGAGGACGATGGTGAATCGTATGC	" "
LYS21-B	TGCTTAGGATCCCCTGGTATTACGGGAAATGC	" "
*LYS21*p		" "
*LYS21*t		" "
RTG2-X	TAAGCACTCGAGCGAGCTCAATAAGTGATCCG	" "
RTG2-B	TGCTTAGGATCCAAGTCCGTTGGTATCATCGG	" "
*RTG2*p	TGCTAACTTCTGCTGGGAAG	" "
*RTG2*t	CGTCACGAATAAATCCCGTG	" "
pCUP1p	ATTAATGCAGCTGGCACGAC	" "
pCUP1t	TCTTTTCCGCTGAACCGTTC	" "
Plasmids sequencing		
*LYS20*f	AGAGGTATATCCACTTCGCC	This study
*LYS20*g	AGTTCCGGTACATCGCTGTC	" "

*LYS21*f	GCACAAGATCAGAGACATCG	" "
*LYS21*g	TGCCTTTTTAGAAACATGCCC	" "
*RTG2*f	CGATAGTGATACCGAGACTG	" "
*RTG2*g	GCACGCCAATTTTAACCCTC	" "
pCUP1p	ATTAATGCAGCTGGCACGAC	" "
pCUP1t	TCTTTTCCGCTGAACCGTTC	" "
qRT-PCR		
*ACT1*f	TCCCAGGTATTGCCGAAAGAATGC	(Starovoytova *et al*., 2013)
*ACT1*r	GCCAAGATAGAACCACCAATCCAGAC	" "
*CIT2*f	GGTATATGGTGGTATGAGAGGTATTC	" "
*CIT2*r	CTTCTGGTAGTGGTTGTGAGC	" "
*DLD3*f	TGTCAAGTGGGCGGTGTAG	" "
*DLD3*r	AGTCATAACCAGTATTGTCCTTCC	" "
