# Supplementary figures and images for: Activation of the yeast Retrograde Response pathway by adaptive laboratory evolution with S-(2-aminoethyl)-L-cysteine reduces ethanol and increases glycerol during winemaking
Source: Microb Cell Fact. 2024 Aug 20;23:231. doi: 10.1186/s12934-024-02504-z (PMC11337681; doi:10.1186/s12934-024-02504-z)

Additional file 5.

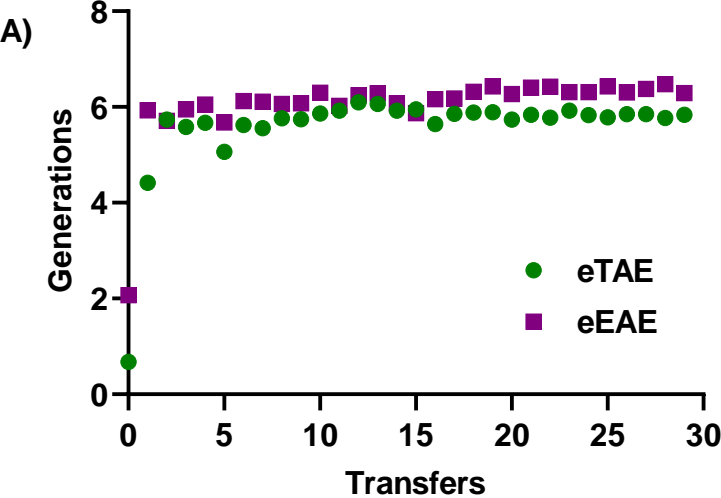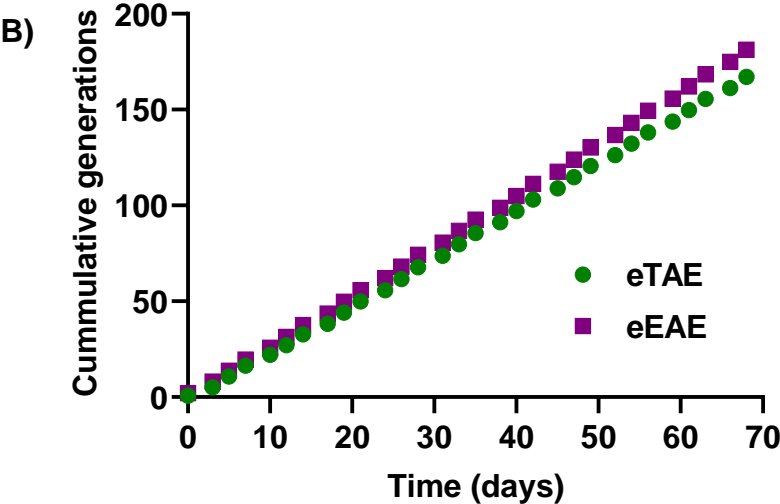

C)

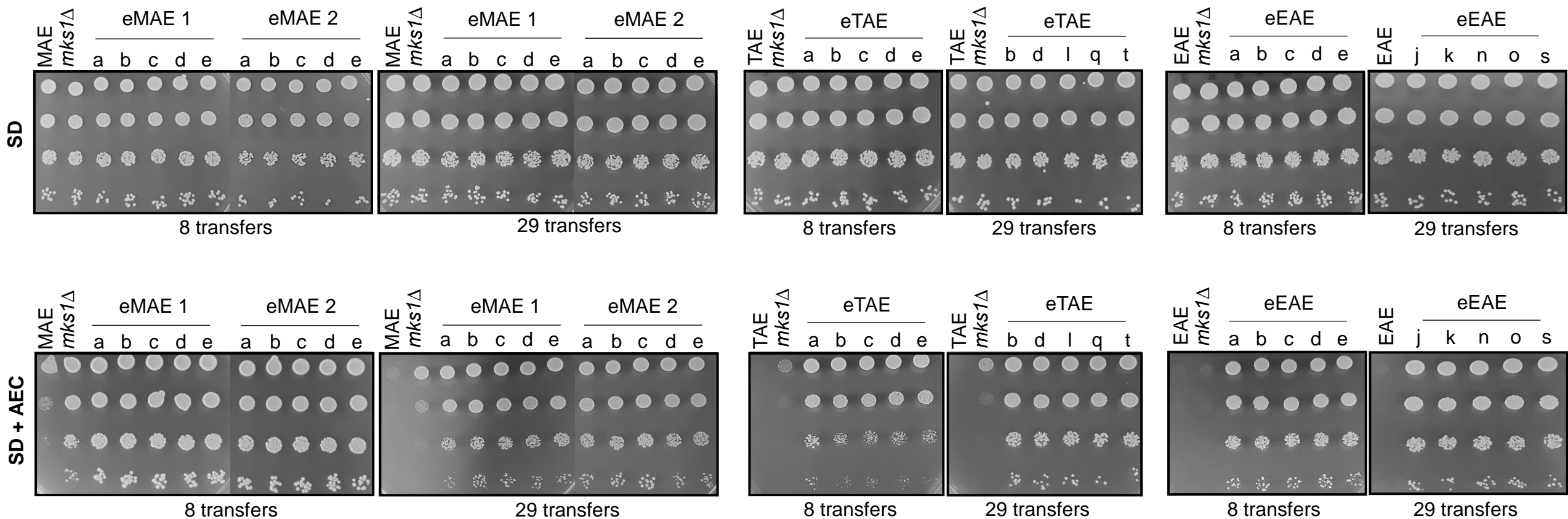

Supplement: Supplementary file 5 — Additional file 5: Monitoring adaptive laboratory evolution experiments of the TAE and EAE strains and thialysine-resistance characterization of all individual clones isolated after 8 and 29 transfers from the MAE, TAE and EAE evolved populations. (A) Generations obtained in each of the transfers performed during the directed evolution of the TAE and EAE strains and (B) accumulated generations throughout the experiment. The black arrows indicate 8 and 29 transfers, the time points at which individual clones were isolated from the evolved population. (C) Spot growth analysis to test the tolerance of each clone isolated from MAE, TAE and EAE evolutions after 8 and 29 transfers. [file 12934_2024_2504_MOESM5_ESM.pdf]

Additional file 7.

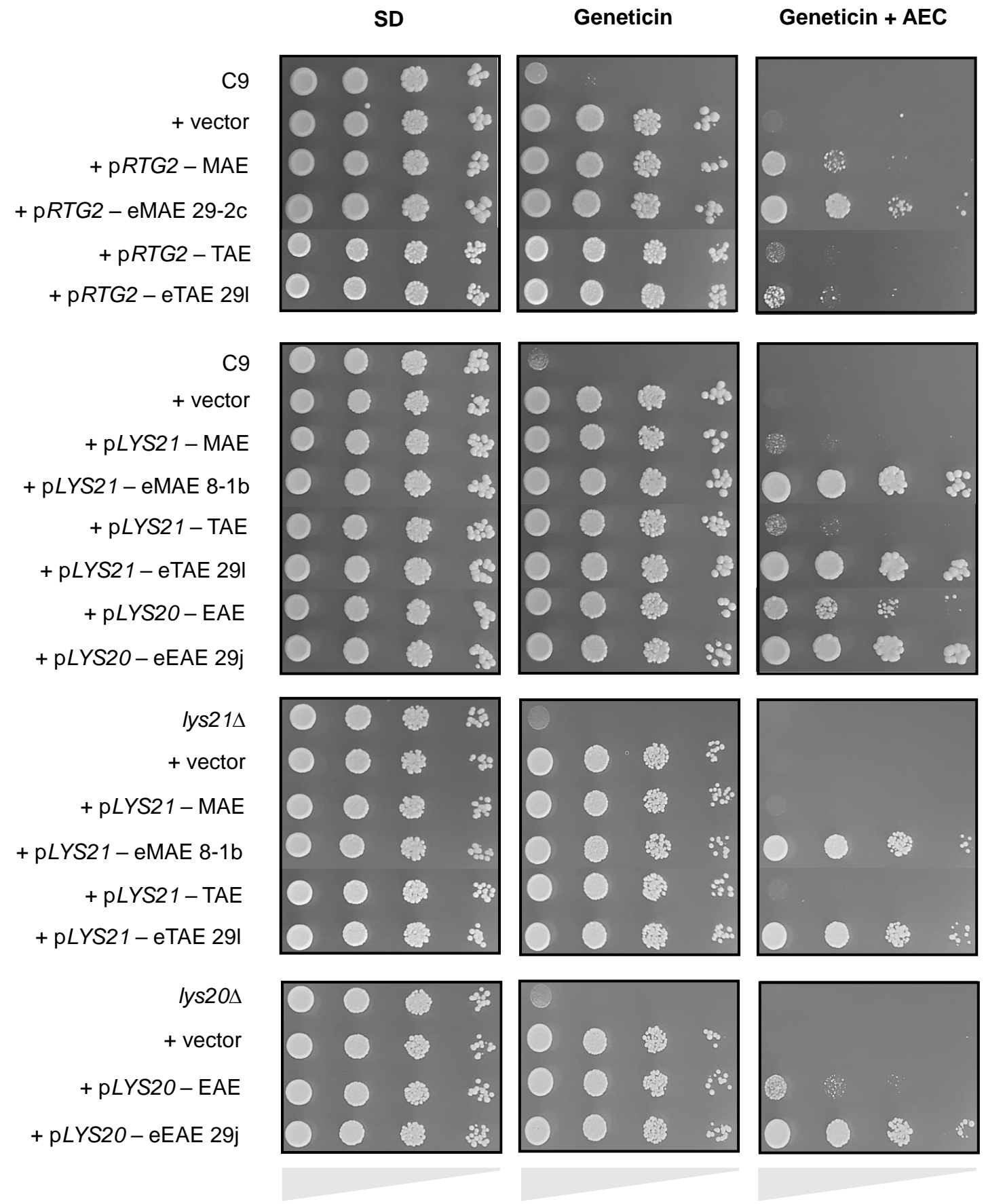

Supplement: Supplementary file 7 — Additional file 7: Involvement of mutations identified in the RTG2, LYS20 and LYS21 genes on the thialysine resistance phenotype. Spot growth analysis of C9, C9 lys20∆ and C9 lys21∆ mutants containing the empty vector (+ vector), the RTG2, LYS20 and LYS21 alleles of the parental strains, or the alleles carrying the mutations identified in eMAE 8-1b, eMAE 29-2c, eTAE 29l and eEAE 29j evolved strains. A 5-μl volume of each serially diluted culture (from 10-1 to 10-4) was spotted onto SD (untreated) plates or SD plates containing 200 mg/L geneticin with or without 35 mg/L AEC. All plates were incubated at 30°C for 48h. [file 12934_2024_2504_MOESM7_ESM.pdf]
