# Supplementary material for: Activation of the yeast Retrograde Response pathway by adaptive laboratory evolution with S-(2-aminoethyl)-L-cysteine reduces ethanol and increases glycerol during winemaking
Source: Microb Cell Fact. 2024 Aug 20;23:231. doi: 10.1186/s12934-024-02504-z (PMC11337681; doi:10.1186/s12934-024-02504-z)
Supplement: Supplementary file 6 — Additional file 6: Values of the main metabolites and residual sugars at the end of fermentation. Glycerol, acetic acid and ethanol levels of the isolated evolved clones after 8 and 29 transfers, that registered significative differences compared to the parental strain at the end of fermentations in sterilised natural red grape must (Bobal variety) with an initial concentration of reducing sugars of 250 g/L. The final values for residual sugars have also been indicated. Fermentation was carried out in triplicate, and the average and standard deviations are provided. Statistical differences (* p < 0.05, Student’s t-test) between the evolved clones and their parental strains are shown. [file 12934_2024_2504_MOESM6_ESM.docx]

**Additional file 6. Values of the main metabolites and residual sugars at the end of fermentation.** Glycerol, acetic acid and ethanol levels of the isolated evolved clones, after 8 and 29 transfers, that registered significative differences compared to the parental strain at the end of fermentations in sterilised natural red grape must (Bobal variety) with an initial concentration of reducing sugars of 250 g/L. The final values for residual sugars have also been indicated. Fermentations were carried out in triplicate, and average and standard deviations are provided. Statistical differences (* p < 0.05, Student’s t-test) between the evolved clones and their parental strains are shown.

|  | **Glycerol (g/L)** | | | **Acetic acid (g/L)** | | | **Ethanol (g/100mL)** | | | **Residual sugars (g/L)** | | |
| --- | --- | --- | --- | --- | --- | --- | --- | --- | --- | --- | --- | --- |
|  | **mean** | **±** | **std desv** | **mean** | **±** | **std desv** | **mean** | **±** | **std desv** | **mean** | **±** | **std desv** |
| MAE | 5.05 | ± | 0.59 | 13.72 | ± | 0.21 | 0.15 | ± | 0.03 | 0.13 | ± | 0.48 |
| eMAE 8-1b | 6.97 | ± | 0.26 * | 13.18 | ± | 0.27 * | 0.05 | ± | 0.01 * | 0.00 | ± | 0.29 |
| eMAE 8-1e | 6.91 | ± | 0.28 * | 13.83 | ± | 0.22 | 0.05 | ± | 0.02 * | 0.13 | ± | 1.73 |
| eMAE 8-2d | 6.89 | ± | 0.36 * | 13.88 | ± | 0.16 | 0.05 | ± | 0.01 * | 0.00 | ± | 0.91 |
| eMAE 8-2e | 7.47 | ± | 0.54 * | 15.28 | ± | 1.23 * | 0.08 | ± | 0.03 * | 0.24 | ± | 6.01 |
| MAE | 7.13 | ± | 0.64 | 13.55 | ± | 0.41 | 0.42 | ± | 0.01 | 1.60 | ± | 1.54 |
| eMAE 29-1e | 9.77 | ± | 0.55 * | 13.54 | ± | 0.63 | 0.07 | ± | 0.03 * | 2.05 | ± | 0.11 |
| eMAE 29-2a | 9.51 | ± | 0.55 * | 12.71 | ± | 0.41 * | 0.26 | ± | 0.02 * | 0.45 | ± | 0.29 |
| eMAE 29-2b | 10.14 | ± | 0.60 * | 13.49 | ± | 0.80 | 0.06 | ± | 0.02 * | 1.73 | ± | 1.86 |
| eMAE 29-2c | 13.18 | ± | 2.04 * | 12.30 | ± | 0.42 * | 0.06 | ± | 0.01 * | 0.06 | ± | 0.11 |
| TAE | 8.53 | ± | 0.31 | 13.04 | ± | 0.61 | 0.29 | ± | 0.01 | 0.50 | ± | 2.17 |
| eTAE 8a | 10.76 | ± | 0.15 * | 12.55 | ± | 0.42 | 0.22 | ± | 0.02 * | 1.86 | ± | 0.63 |
| TAE | 8.53 | ± | 0.15 | 13.54 | ± | 0.06 | 0.30 | ± | 0.01 | 1.44 | ± | 1.25 |
| eTAE 29b | 15.86 | ± | 0.10 * | 12.69 | ± | 0.18 * | 0.13 | ± | 0.01 * | 1.46 | ± | 2.53 |
| eTAE 29d | 16.51 | ± | 0.51 * | 12.37 | ± | 0.18 * | 0.12 | ± | 0.01 * | 2.40 | ± | 0.36 |
| eTAE 29l | 16.92 | ± | 0.48 * | 11.66 | ± | 0.21 * | 0.12 | ± | 0.00 * | 1.75 | ± | 0.65 |
| eTAE 29q | 15.85 | ± | 0.17 * | 11.55 | ± | 0.51 * | 0.11 | ± | 0.01 * | 1.91 | ± | 0.18 |
| eTAE 29t | 16.60 | ± | 0.26 * | 11.98 | ± | 0.31 * | 0.12 | ± | 0.02 * | 4.29 | ± | 2.30 |
| EAE | 7.97 | ± | 0.05 | 13.82 | ± | 0.23 | 0.20 | ± | 0.01 | 1.81 | ± | 1.10 |
| eEAE 29j | 9.14 | ± | 0.24 * | 13.84 | ± | 0.20 | 0.15 | ± | 0.00 * | 0.63 | ± | 0.83 |
| eEAE 29o | 14.86 | ± | 0.10 * | 13.59 | ± | 0.09 | 0.15 | ± | 0.00 * | 1.78 | ± | 2.55 |
